# Redescription of the soft-shell turtle *Rafetus bohemicus* (Testudines, Trionychidae) from the Early Miocene of Czechia

**DOI:** 10.7717/peerj.15658

**Published:** 2023-07-27

**Authors:** Milan Chroust, Martin Mazuch, Martin Ivanov, David M. Alba, Àngel H. Luján

**Affiliations:** 1 Charles University, Institute of Geology and Palaeontology, Prague, Czech Republic; 2 Institute of Geology of the Czech Academy of Sciences, Department of Paleobiology and Paleoecology, Prague, Czech Republic; 3 Masaryk University, Department of Geological Sciences, Brno, Czech Republic; 4 Institut Català de Paleontologia Miquel Crusafont, Universitat Autònoma de Barcelona, Cerdanyola del Vallès, Barcelona, Spain

**Keywords:** *Trionyx*, *Rafetus*, Bohemia, Most Basin, Břešt’any, Burdigalian, Trionychinae, Czech Republic

## Abstract

The taxonomy of the soft-shell turtle *Rafetus bohemicus* (Liebus, 1930), family Trionychidae, subfamily Trionychinae, is revised based on new and previously mentioned material (including the type material) from the Early Miocene (Burdigalian, MN 3) sites of the Most Basin, Czechia. Given that the diagnosis was so far based only on plastral elements, here we focused on the cranial material and combined our study with previously published data on postcranial elements. 3D models of the skulls derived from CT scans allow us to provide the first complete skull description of *R. bohemicus*, including several new cranial diagnostic characters of the species. Our results not only enable the distinction of the trionychid genera *Trionyx* and *Rafetus*, both recorded from Central Europe during the Early Miocene, but further allow us to provide an emended diagnosis for *R*.* bohemicus*. We confirm the conclusions of a previous study according to which *Trionyx pontanus*, *T*. *preschenensis*, *T*. *aspidiformis*, and *T*. *elongatus* are *nomina dubia*. *R. bohemicus* from Břešt’any (MN 3) represents the oldest record of this genus in Europe as well as the oldest occurrence of the genus.

## Introduction

The soft-shell turtles (Trionychidae) are freshwater turtles characterized by reduced carapace and plastron elements covered by skin as well as paddle-like limbs (Meylan, 1987; Delfino et al., 2010; Vitek & Joyce, 2015). Trionychids originated in Asia during the Early Cretaceous and expanded into North America and Europe by the Late Cretaceous. They reached Africa and Australasia during the Paleogene and, for a short period, they also inhabited Central and South America during the Neogene (Nessov, 1995; Sánchez-Villagra et al., 2004; Head, Aguilera & Sánchez-Villagra, 2006; Vitek & Danilov, 2010; Cadena et al., 2012; Scheyer, Mörs & Einarsson, 2012; Hirayama, Isaji & Hibino, 2013; Li, Joyce & Liu, 2015; Li et al., 2015; Vitek & Joyce, 2015; Georgalis & Joyce, 2017; Li et al., 2017; Georgalis, 2021; Selvatti et al., 2023). Extant soft-shell turtles are distributed in Asia, North America, Africa, Australia and occasionally also in the Dodecanese Islands of southeastern Europe (Engstrom, Shaffer & McCord, 2004; Georgalis & Joyce, 2017; Li et al., 2017).

The Miocene European fossil record of soft-shell turtles is very rich and more than 40 different trionychid nominal species were erected. Five different species were described from the Most Basin (NW Bohemia, Czechia), of which four species are from a single fossil site of Břešt’any (Laube, 1900; Liebus, 1930). According to a recent taxonomic revision (Georgalis & Joyce, 2017), only two Miocene species can be considered taxonomically valid: *Rafetus bohemicus* (Liebus, 1930), the species closest to the extant species of this genus; and *Trionyx vindobonensis* (Peters, 1855), close to the extant *Trionyx triunguis* (Forskål, 1775). In addition, Georgalis et al. (2020) described material from the Late Miocene of southern Italy of the otherwise Pliocene species *Trionyx pliocenicus* (Fucini, 1912), thereby increasing the number of valid Miocene European species to three. The record of *R. bohemicus* is restricted to the Burdigalian of Czechia (Liebus, 1930; Georgalis & Joyce, 2017), whereas *T. vindobonensis* ranges from the Aquitanian to the Tortonian and is otherwise recorded from Austria, France, and Germany (Georgalis & Joyce, 2017). According to Georgalis & Joyce (2017), *R. bohemicus* mainly differs from *T. vindobonensis* in having a highly reduced costal VIII and lacking a developed callosity in the xiphiplastron.

Here, we revise the taxonomy of published trionychids from the Most Basin based on all available remains, including their type material. The skull of *R. bohemicus* is described in detail based on remains from Břešt’any, including new diagnostic characters that are incorporated into an emended diagnosis. The complex taxonomic and nomenclatural history of soft-shell turtles from the Most Basin is discussed in detail.

## Age and Geological Background

**Figure 1:**
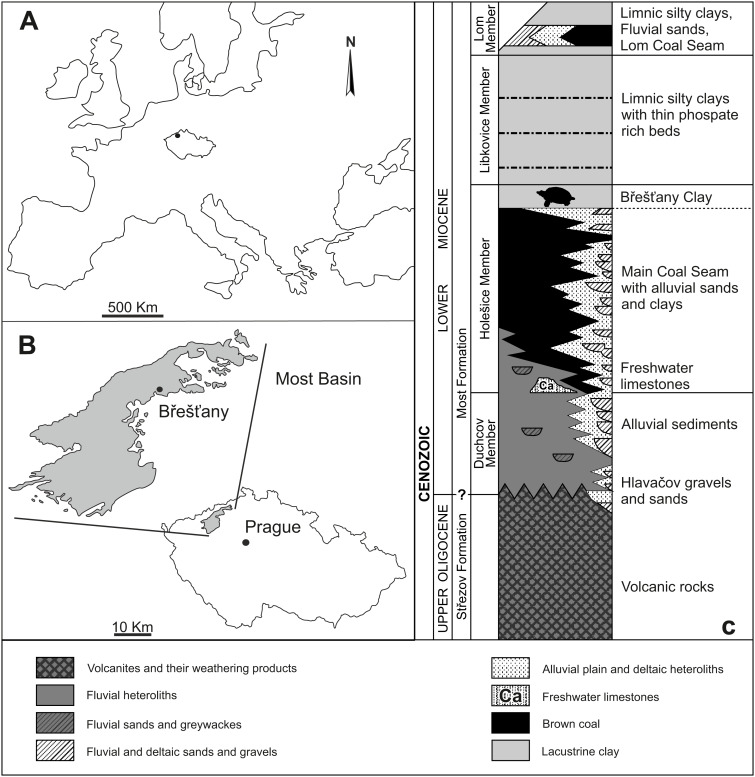
Geographical location and general context of the Most Basin showing the location of the study area. (A) Location map of Czechia. (B) Position of Břešt’any within the Most Basin. (C) Stratigraphic column of the Most Basin. Modified from Mach et al. (2017).

Břešt’any is one of the richest fossil sites from the European Early Miocene (Burdigalian; Aguilar, Legendre & Michaux, 1997). It is renowned for its fossil plant remains (for further details, see Teodoridis & Kvaček, 2006), but vertebrates are preserved as well. The site of Břešt’any (also called Preschen in old literature) was located between the towns of Břešt’any, Břežánky, and Jenišův Újed, ∼3 km NW from the city of Bílina (Dvořák et al., 2010). From a geological viewpoint, it was situated on the northeastern margin of the Most Basin (NW Bohemia, Czechia; Figs. 1A–1B), which is the largest basin along the Ohře/Eger Graben, a European Cenozoic Rift System (Kvaček et al., 2004; Rajchl et al., 2009; Matys Grygar & Mach, 2013; Luján et al., 2019). The Břešt’any Clay, a specific horizon of limnic ceramic clays belonging to the Holešice Member (Most Formation), was the main object of mining, along with coal deposits, during the last two centuries. However, the whole area was destroyed during the 1990s due to the expanding mining of the underlying Main Coal Seam at Bílina mine (Dvořák et al., 2010; Matys Grygar & Mach, 2013). The oldest geological unit of the Most Basin is composed by Oligocene volcanic rocks of the Střezov Formation (Fig. 1C). It is overlain by the Miocene fluvial-lacustrine deposits of the Most Formation (Rajchl et al., 2009; Mach et al., 2014). A large peat accumulation of the swamp area, shortly after its deposition, was transformed into the Main Coal Seam and covered mainly by lacustrine sediments (Matys Grygar et al., 2014; Matys Grygar et al., 2017b). The final flooding event, in fact, formed the Břešt’any Clay horizon (Matys Grygar et al., 2017a), which is a fine mudstone rich in kaolinite and fossils as well as with occasional cementation of the siderite (Matys Grygar & Mach, 2013; Matys Grygar et al., 2017b). From a sedimentological point of view, the Břešt’any Clay represents an isolated lateral lacustrine sedimentation, parallel to the development of Bílina delta, subsequently followed by a lake transgression in the entire Bílina area (Rajchl et al., 2009; Matys Grygar & Mach, 2013). A short overlying horizon with a thin coal seam layer can be discerned, which shows an interval of water table decrease and the re-development of the swampy area, *i.e.,* Lom Coal Seam (Matys Grygar et al., 2017a). Younger deposits in the Most Basin are beyond the scope of this work. The site of Břešt’any is slightly younger than the Ahníkov fossil site, dated to the Burdigalian (MN 3 Mammal Neogene unit) based on micromammal assemblages (Fejfar, 1989; Fejfar & Kvaček, 1993; Fejfar, Dvořák & Kadlecová, 2003; Fejfar & Sabol, 2005; Van den Hoek Ostende & Fejfar, 2006; Van den Hoek Ostende & Fejfar, 2015). The chemostratigraphic border between the Holešice and Libkovice members, where the Břešt’any Clay is located, has been dated to ∼17.5 Ma (Matys Grygar et al., 2017b; Ekrt, Novotný & Přikryl, 2022).

## Materials and Methods

The anatomical nomenclature employed in this paper for the skulls is mainly based on Gaffney (1972) and Gaffney (1979), whereas the shell anatomical nomenclature follows Zangerl (1969). For the alpha-taxonomy of extant and extinct trionychid species, we follow Georgalis & Joyce (2017) and Joyce et al. (2021). However, note that these authors used unranked phylogenetic nomenclature, whereas we prefer to use Linnean ranks.

Skeletal remains are preserved in limnic clay or siderite concretions. Apart of the bones in siderite concretions, the material mainly creates only its imprints. Thus, the specimens are very fragile and therefore a mechanic extraction was impossible. Three selected skulls (*i.e.,* NMP Pv 11668, MMUL 1037/G 12917 and RMT PA 1310) were scanned at the General University Hospital in Prague, using an X-ray CT scanner (model iCT Brilliance 256; Philips Healthcare, Eindhoven, Netherlands). We used different scanning parameters to get the best possible images: from 80 kV and 45 mAs up to 140 kV and 205 mAs; slice thickness of 0.9 mm, with 50% overlap; and effective slice thickness of 0.45 mm. Subsequently, we selected the images with the least noise (*i.e.,* 140 kV and 205 mAs) and post-processed the data with the software Philips IntelliSpace Portal v.10.1 to derive the 3D models. The matrix was mostly removed, but the sutures were not visible despite ourselves adjusting thresholds manually. However, only the virtual models of NMP Pv 11668 and MMUL 1037/G 12917 bring useful information. According to these preliminary results, the best-preserved skull NMP Pv 11668 was scanned for the second time with a more powerful X-ray CT scanner: TORATOM (Twinned Orthogonal Adjustable Tomograph) at the Centre Telč, Institute of Theoretical and Applied Mechanics of the Czech Academy of Sciences. The parameters used this time were the following: 230 kV and 250 mAs; 2,400 slices; and a reached resolution of 60 µm per voxel. VG Studio Max 3.2 was used for the reconstruction and visualization of the resulting 3D model, which was slightly better than those derived from the medical CT data. All 3D reconstructions are uploaded on Morphosource, CT image series (*Rafetus bohemicus* NMP Pv 11668, Media ID: 000469442, DOI: 10.17602/M2/M469442), the Mesh CT file (*Rafetus bohemicus* NMP Pv 11668, Media ID: 000474314, DOI: 10.17602/M2/M474314) and the video file (*Rafetus bohemicus* NMP Pv 11668, Media ID: 000459259, DOI: 10.17602/M2/M459259 and Supplemental Material 1). All fossil turtle material reported in this paper is listed in Table 1. As for the trionychid material stored in the National Museum in Prague, we used the catalog number plus access number. To avoid confusion, material housed at Ústí nad Labem was used with historical and current valid numbers. Extant species included in the comparative sample are housed at NHMUK (*Rafetus swinhoei* NHMUK 1947.3.6.13), IEBRH (*Rafetus swinhoei* NQT85), NHMW (*Rafetus euphraticus* NHMW 130, NHMW 93.10.14.1, NHMW 1856 and *Trionyx triunguis* NHMW 159, NHMW 20993) and IPUW (*Trionyx vindobonensis* IPUW 1897 IV).

### Systematic Paleontology

**Table:** 

TESTUDINES Batsch, 1788
CRYPTODIRA Cope, 1868
TRIONYCHIDAE Bell, 1828
TRIONYCHINAE Lydekker, 1889

Genus *Trionyx* Geoffroy Saint-Hilaire, 1809

**Table 1:** List of fossil specimens included in this study.

**Catalogue no.**	**Anatomical** **description**	**Locality**	**Material**	**References**	**Figure**
RMGM G/pa77a	Partial carapace (imprint of RMGM G/pa77b)	Lom u Mostu	Syntype of *Trionyx pontanus*	Laube (1896)	2A–B
RMGM G/pa77b	Partial carapace	Lom u Mostu	Syntype of *Trionyx pontanus*	Laube (1896)	2C–D
Without number (currently lost)	Partial carapace (imprint of NMP Pv 12301)	Louka u Litvínova	Syntype of *Trionyx pontanus*	Laube (1896)	3A–B
NMP Pv 12301	Partial carapace and hypoplastron	Louka u Litvínova	Syntype of *Trionyx pontanus*	Laube (1896)	3C–D
CUP CHMHZ-GL-0001	Almost complete carapace and hypoplastron	Břešt’any	Syntype of *Trionyx preschenensis*	Laube (1900)	4A–B
NMP 20205/Pb 72	Partial carapace (imprint of CMEHCUP CHMHZ-GL-0001)	Břešt’any	Syntype of *Trionyx preschenensis*	Laube (1900)	4C–D
NMP 36675/Pb 73	Partial carapace	Břešt’any	Holotype of *Trionyx aspidiformis*	Laube (1900)	5A–B
RMT PA 1308/1	Partial carapace, pectoral girdle fragment and right femur	Břešt’any	Referred material of *Trionyx aspidiformis*	Liebus (1930)	6A–B
RMT PA 1308/2	Partial carapace and femur (imprint of RMT PA 1308/1)	Břešt’any	Referred material of *Trionyx aspidiformis*	Liebus (1930)	6C–D
NMP 1488/Pb 4	Almost complete carapace and hypoplastron	Břešt’any	Syntype of *Trionyx elongatus*	Liebus (1930)	7A–B
MMUL 129/G 12911	Complete nuchal	Břešt’any	Syntype of *Trionyx elongatus*	Liebus (1930)	7C–D
NMP Pv 11668	Partial skull	Břešt’any	Referred material of *Trionyx bohemicus*		8A
MMUL 1442/G 10193	Partial skull	Břešt’any	Syntype of *Trionyx bohemicus*	Liebus (1930)	10A–B
MMUL 1443/G 12926	Skull (imprint of MMUL 1442/G 10193)	Břešt’any	Syntype of *Trionyx bohemicus*	Liebus (1930)	10C
RMT PA 1309	Partial skull	Břešt’any	Referred material of *Trionyx bohemicus*		11A–B
RMT PA 1310	Partial skull (imprint of the RMT PA 1309)	Břešt’any	Referred material of *Trionyx bohemicus*		11C
MMUL 1049/G 10196	Partial skull	Břešt’any	Syntype of *Trionyx bohemicus*	Liebus (1930)	11D–E
MMUL 1037/G 12917	Partial skull and xiphiplastron	Břešt’any	Syntype of *Trionyx bohemicus*	Liebus (1930)	12A–B
MMUL 1042/G 12922	Partial skull and xiphiplastron (imprint of MMUL 1037/G 12917)	Břešt’any	Syntype of *Trionyx bohemicus*	Liebus (1930)	12C
MMUL 1048/G 10194	Partial skull	Břešt’any	Syntype of *Trionyx bohemicus*	Liebus (1930)	12D–E
MMUL 633/2ab/G 12908/G 12941	Partial carapace	Břešt’any	Syntype of *Trionyx bohemicus*	Liebus (1930)	13A–B
MMUL 633/1/*G* 12910	Partial carapace (imprint of MMUL 633/2ab/G 12908/G 12941)	Břešt’any	Syntype of *Trionyx bohemicus*	Liebus (1930)	13C–D
NMP 1485a/Pb 2a	Partial carapace	Břešt’any	Syntype of *Trionyx bohemicus*	Liebus (1930)	14A–B
NMP1485b/Pb 2b	Partial carapace (imprint of NMP Pb 2a)	Břešt’any	Syntype of *Trionyx bohemicus*	Liebus (1930)	14C–D
NMP P/9640(4)/Pv 12303	Epiplastron	Břešt’any	Syntype of *Trionyx bohemicus*	Liebus (1930)	15A–B
MMUL 1444/G 12927	Almost complete entoplastron	Břešt’any	Syntype of *Trionyx bohemicus*	Liebus (1930)	15A–B
MMUL I/G 1486/G 12939	Almost complete hyoplastron	Břešt’any	Syntype of *Trionyx bohemicus*	Liebus (1930)	15A–B
MMUL 1035/G 12915	Hypoplastron	Břešt’any	Syntype of *Trionyx bohemicus*	Liebus (1930)	15A–B
MMUL 1038/G 12918	Almost complete xiphiplastron	Břešt’any	Syntype of *Trionyx bohemicus*	Liebus (1930)	15A–B
MMUL 1452/G 12934	Partial cervical III	Břešt’any	Syntype of *Trionyx bohemicus*	Liebus (1930)	16A
MMUL 1451/G 12933	Partial cervical IV	Břešt’any	Syntype of *Trionyx bohemicus*	Liebus (1930)	16B
NMP Pv 12302	Partial cervical IV	Břešt’any	Syntype of *Trionyx bohemicus*	Liebus (1930)	16C
NMP Pb 51	Partial cervical VI	Břešt’any	Syntype of *Trionyx bohemicus*	Liebus (1930)	16D
MMG CST 621	Complete cervical VIII	Břešt’any	Referred material of *Trionyx bohemicus*	Laube (1901)	16E
MMUL 631/G 12912	Almost complete pectoral girdle	Břešt’any	Syntype of *Trionyx bohemicus*	Liebus (1930)	17A–B
MMUL 1461/G 12937	Coracoid	Břešt’any	Syntype of *Trionyx bohemicus*	Liebus (1930)	17C
RMT PA 1684	Almost complete humerus	Břešt’any	Referred material of *Trionyx bohemicus*		17D–E
RMT PA 1686	Partial pelvic girdle	Břešt’any	Referred material of *Trionyx bohemicus*		18A–B
RMT PA 1683	Right femur	Břešt’any	Referred material of *Trionyx bohemicus*		18C–D
RMT PA 1693	Left femur	Břešt’any	Referred material of *Trionyx bohemicus*		18E

Type species: *Testudo triunguis* Forskål, 1775.

*Trionyx pontanus* Laube, 1895

*nomen dubium*

(Figs. 2–3)

Taxonomic history: see Georgalis & Joyce (2017: p. 174).

**Materials.** RMGM G/pa77 (syntype), a nearly complete carapace and its imprint (Laube, 1896; pls. 1–2, figures without numbers). Type locality: Evžen (Eugen) Mine, Lom u Mostu (Bruch bei Brüx), Czechia; NMP Pv 12301 (syntype) rather complete carapace and its imprint (currently lost) with the left hypoplastron (Laube, 1896; pls. 3–4).

**Type locality.** Pavel I Mine (Paulschacht), Louka u Litvínova (Wiese bei Leutensdorf), Czechia.

### Description

**Figure 2:**
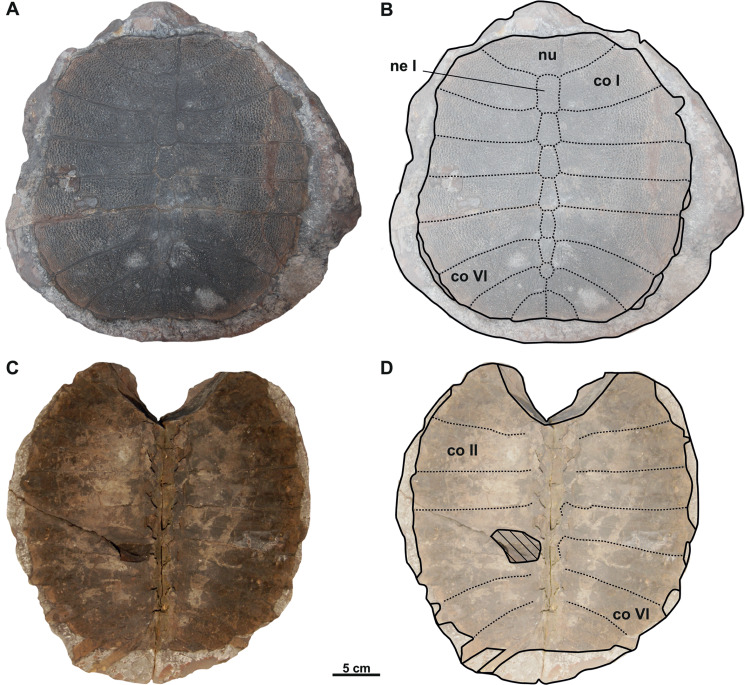
Photographs and schematic drawings of syntype material of *Trionyx pontanus* from Lom u Mostu (A–D). Imprint of the carapace RMGM G/pa77a in visceral (A–B) views. Original of the carapace RMGM G/pa77b in dorsal (C–D) views. Abbreviations: co I, costal I; co II, costal II; co VI, costal VI; ne I, neural I; nu, nuchal.

**Figure 3:**
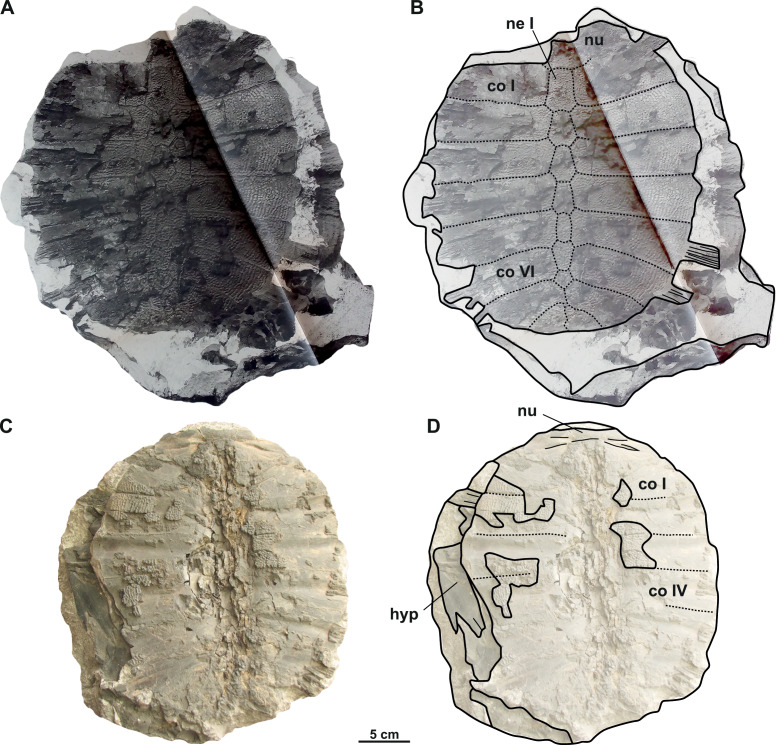
Photographs and schematic drawings of syntype material of *Trionyx pontanus* from Louka u Litvínova (A–D). Imprint of the carapace (without number, currently lost) in visceral (A–B) views (reproduced from Laube, 1896). Original carapace NMP Pv 12301 in dorsal (C–D) views. Abbreviations: co I, costal I; co IV, costal IV; co VI, costal VI; hyp, hypoplastron; ne I, neural I; nu, nuchal.

Both syntypes (RMGM G/pa77a–b and NMP Pv 12301; Figs. 2A–2D, 3C–3D) belong to a medium-sized trionychid, with a carapace length of about 33 cm and 28 cm, respectively. RMGM G/pa77a is much better preserved than NMP Pv 12301, in which the nuchal plate is only partially preserved and several costal plates are highly eroded (Figs. 3A–3D). The sculpturing pattern covers all the carapace and consists of small tubercles at the neural region and the most medial area of costal plates, as well as tubercles and ridges at the lateral margins of the carapace. Both specimens lack the preneural plate and suprascapular fontanelles (Figs. 2A–2B, 3A–3B). The nuchal is wider than long and is placed anterior to the disc formed by the costals. Seven neurals are preserved in total. Neural I is the largest plate and is subhexagonal with the shorter sides behind. Neurals II–IV are roughly similar in size and display the same shape as neural I. However, neural V marks the reversal in the arrangement of neurals, displaying a subrectangular shape (Figs. 2A–2B, 3A–3B). Neural VI is hexagonal, with the shorter sides behind, whereas neural VII is a small pentagonal element, which only partially separates costal VII along the midline. Eight costals are preserved: the first six are rectangular, costal VII is trapezoidal and costal VIII is almost triangular (Figs. 2A–2B, 3A–3B). The anterior and posterior margins of costal I are concave, whereas the sutures of costals III–IV are more or less perpendicular to the axis plane. In all preserved carapaces, costal IV is the widest plate: from costal V onward, the anterior and posterior edges of costals become convex. As seen in all trionychids, suprapygals, pygal and peripheral bones are absent. The preserved portion of the left hypoplastron in NMP Pv 12301 only enables the evaluation of its posterolateral hypoplastral process, which is double (Figs. 3C–3D).

*Trionyx preschenensis* Laube, 1900

*nomen dubium*

(Figs. 4A–4D)

**Figure 4:**
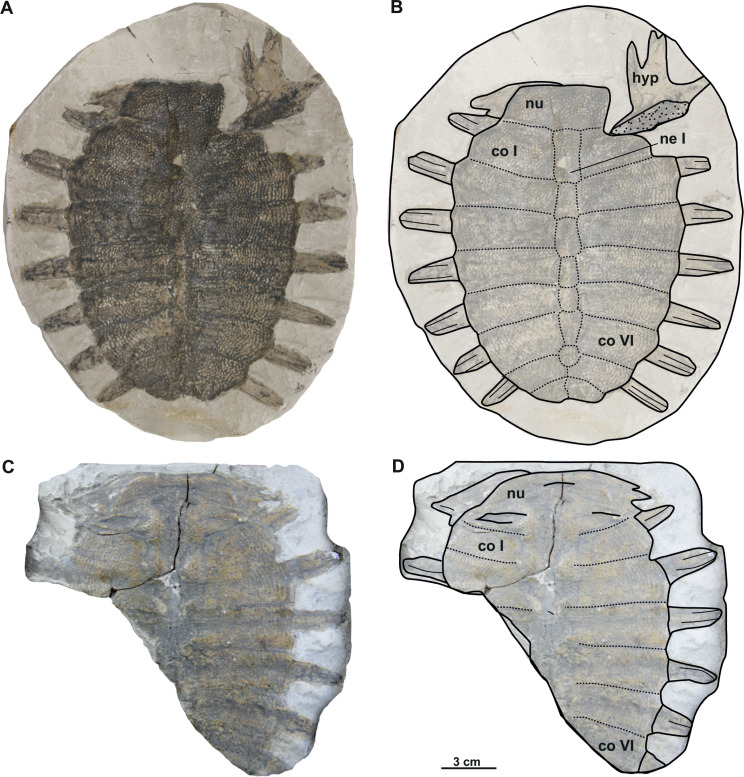
Photographs and schematic drawings of the syntype material of *Trionyx preschenensis* from Břešt’any (A–D). Original (CUP CHMHZ–GL–0001) and its imprint (NMP 20205/Pb 72) of a carapace with the hypoplastron (syntypes) of *T. preschenensis* in dorsal (A–B) and visceral (C–D) views. Abbreviations: co I, costal I; co VI, costal VI; hyp, hypoplastron; ne I, neural I; nu, nuchal.

Taxonomic history: see Georgalis & Joyce (2017: p. 174).

**Material.** CUP CHMHZ–GL–0001 (syntype), an almost complete carapace with a partial right hypoplastron (Laube, 1900; pl. 2). NMP 20205/Pb 72 (syntype), the imprint of CUP CHMHZ–GL–0001 (Laube, 1900; pl. 3, Fig. 1).

**Type locality.** Břešt’any (Preschen), Czechia.

### Description

The syntype of *Trionyx preschenensis* (CUP CHMHZ–GL–0001) has a carapace 17.8 cm long and 13.6 cm wide, not considering the free part of the dorsal ribs (Figs. 4A–4B). The external surface of the carapace is entirely ornamented by ridges and tubercles and has no scutes. The nuchal plate is four times wider than long. This specimen lacks suprascapular fontanelles as evidenced by the continuous sutures between the nuchal, neural I and costals I (Figs. 3A–3B). The preneural plate is absent. The total number of neural plates is seven. However, they are fully sutured to each other, and the sutures cannot be adequately discerned. The specimen has eight pairs of costals, being costal II and III the largest ones. Costals I–VII are subrectangular (wider than long), but costal VIII is rather trapezoidal. It is worth highlighting that the width of the costal VIII is approximately half than that of costal VII. All peripheral, suprapygal and pygal plates are absent. As for plastral elements, only the hypoplastron is preserved (Figs. 4A–4B): it is slender, wider than long, and very reduced medially in anteroposterior direction. The contact with the hypoplastron is osseous (*i.e.,* both hypo- and hyoplastron were sutured together, at least partially). A single and robust medial hypoplastral process is present, which develops perpendicular to the axis plane. However, the posterior hypoplastral process is double and runs in posteromedial direction, being the most medial distal end slightly bilobed (*i.e.,* the contact with the xiphiplastron was ligamentous, instead of osseous). The hypoplastron callosities are well developed in ventral view but restricted along the hyo-hypoplastral suture (Figs. 4A–4B), *i.e.,* at least the lateral and posterior processes are free. It seems that all the callosity is sculptured, instead of concentred in the central portion as in other trionychids.

*Trionyx aspidiformis* Laube, 1900

**Figure 5:**
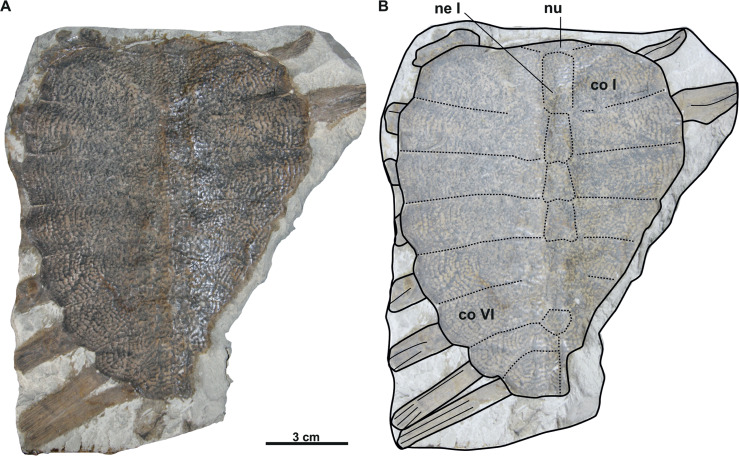
Photographs and schematic drawings of holotype material of *Trionyx aspidiformis* from Břešt’any (A–B). Imprint of a partial carapace NMP 36675/Pb 73 of *T. aspidiformis* in visceral view (A–B). Abbreviations: co I, costal I; co VI, costal VI; ne I, neural I; nu, nuchal.

**Figure 6:**
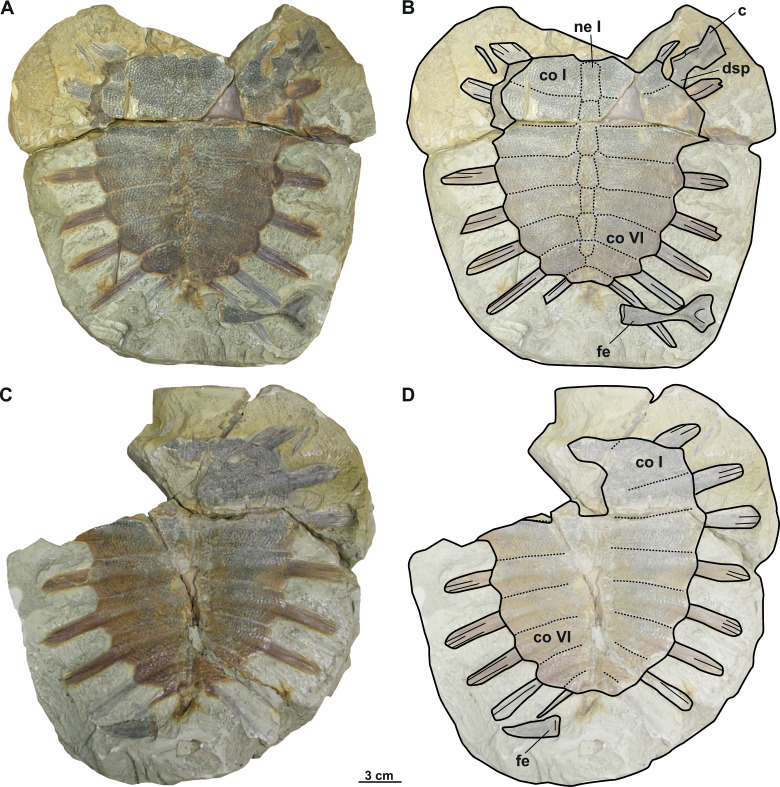
Photographs and schematic drawings of referred material of *Trionyx aspidiformis* from Břešt’any (A–D). Original (RMT PA 1308/1) and its imprint (RMT PA 1308/2) of a carapace with femur and pectoral girdle of *T. aspidiformis* in dorsal (A–B) and visceral (C–D) views. Abbreviations: c, coracoid; co I, costal I; co VI, costal VI; dsp, dorsal scapular process; fe, femur; ne I, neural I.

*nomen dubium*

(Figs. 5A–5B, 6A–6D)

Taxonomic history: see Georgalis & Joyce (2017: p. 156).

**Material.** NMP 36675/Pb 73 (holotype), imprint of a carapace without the posterior left side (Laube, 1900; pl. 3, Fig. 2). RMT PA 1308/1 original of a carapace with left humerus and partially preserved scapula and coracoid (Liebus, 1930; pl. 4, Fig. 1). RMT PA 1308/2, imprint of RMT PA 1308/1 (Liebus, 1930; pl. 4, Fig. 2).

**Type locality.** Břešt’any (Preschen), Czechia.

### Description

The holotype of the second species erected from Břešt’any consists only of an external imprint of a carapace and provides limited information. Furthermore, the dorsoventral compression of the specimen does not enable evaluation of carapace convexity. It is longer than wide and, together with the nuchal plate, strongly eroded. Unlike *T. pontanus* and *T. preschenensis*, NMP 36675/Pb 73 has a pear-shaped carapace, which is ornamented by tubercles and ridges (Figs. 5A–5B). Seven neural plates are relatively well preserved except for the sutures of neural V–VI, which are obliterated (Figs. 5A–5B). The left side of the carapace preserves all costal elements, being costals II–III the widest plates. The lateral extensions of costals VII–VIII (the only ones that are complete) are well developed posterolaterally (Figs. 5A–5B). RMT PA 1308/1 and 1308/2 is the second specimen subsequently referred to *T. aspidiformis* by Liebus (1930). The sculpturing pattern and shape of this shell are identical to those of NMP 36675/Pb 73. It preserves all the neurals, most of the lateral extensions of costals, and a femur and coracoid. The costals are developed in a similar way to NMP 36675/Pb 73, although costal VIII is notably small (*i.e.,* its total width is less than half the width of costal VII). In both specimens, scutes and peripheral, pygal, and suprapygal plates are absent. The preserved appendicular elements of this specimen correspond to the left femur, in which only the diaphysis is slightly hidden (Figs. 6A–6B), and to a partial coracoid. The major and minor trochanters of the femur are well developed medially and not fused into a continuous intertrochanteric crest, so that the intertrochanteric fossa is visible in ventral view (Figs. 6A–6B). The diaphysis is slightly convex instead of straight. The coracoid is flat and fan-shaped, whereas the preserved portion of the scapular process is long (Figs. 6A–6B).

*Trionyx elongatus* Liebus, 1930

*nomen dubium*

(Figs. 7A–7D)

**Figure 7:**
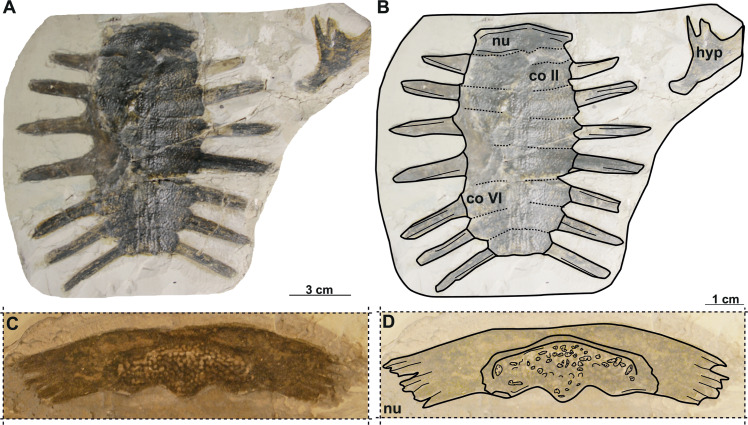
Photographs and schematic drawings of syntype material of *Trionyx elongatus* from Břešt’any (A–D). Almost complete carapace with the hypoplastron NMP 1488/Pb 4 (syntype) of *T. elongatus* in dorsal (A–B) views. Complete nuchal MMUL 129/G 12911 (syntype) of *T. elongatus* in dorsal (C–D) views. Abbreviations: co 2, costal 2; co 6, costal 6; hyp, hypoplastron; nu, nuchal.

Taxonomic history: see Georgalis & Joyce (2017: p. 163).

**Material.** NMP 1488/Pb 4 (syntype), a complete carapace with hypoplastron (Liebus, 1930; pl. 3, Fig. 6); MMUL 129/G 12911 (syntype), a complete nuchal plate (Liebus, 1930; pl. 3, Fig. 7).

**Type locality.** Břešt’any (Preschen), Czechia.

### Description

NMP 1448/Pb 4 (syntype) is an elongated and narrow carapace of a juvenile individual: its total length is 11 cm, and the maximal width is 5.4 cm (taken at the level of costal II). The outer surface is ornamented by a pattern of tubercles and ridges. There are no scutes. All lateral extensions of the costal plates are well developed, except for those of the costals I, which are eroded (Figs. 7A–7B). NMP 1488/Pb 4 lacks the suprascapular fontanelles and the preneural plate. The nuchal plate (MMUL 129/G 12911, syntype) is more than four times wider than long and surrounded by a non-sculptured and large costiform process. The anterior edge of the nuchal is convex in NMP 1488/Pb 4, whereas in MMUL 129/G 12911 (Figs. 7C–7D) it is slightly concave. The posterior edge of the nuchal is medially concave in both specimens (Figs. 7A–7D). The seven neurals series is complete, but the sutures between the neurals are obliterated in NMP 1488/Pb 4: it has eight pairs of costals, the left ones being poorly preserved. Pygal, suprapygal, and peripheral bones are not developed, as in all trionychids. The available hypoplastron is wider than long: both medial and lateral hypoplastral processes are eroded, whereas the posterior one is double and developed posteromedially. Given that this element is preserved in visceral view, it provides no detail regarding the callosity.

**Table:** 

Genus *Rafetus*Gray, 1864
Type species: *Testudo euphratica*Daudin, 1801.

*Rafetus bohemicus* (Liebus, 1930)

(Figs. 8–18)

Taxonomic history: see Georgalis & Joyce (2017: p. 135).

**Material.** The type series is formed by the syntypes described and figured by Liebus (1930): MMUL 633/2ab/G 12908/G 12941, a complete carapace (Liebus, 1930; pl. 1., Fig. 2); MMUL 633/1/G 12910, an imprint of MMUL 633/2ab/G 12908/G 12941 (Liebus, 1930; pl. 1., Fig. 1); NMP 1485a/Pb 2a, an inner imprint of carapace (Liebus, 1930; pl. 2., Fig. 1); NMP 1485b/Pb 2b, an imprint of NMP 1485a/Pb 2a; MMUL 1444/G 12927, an entoplastron (Liebus, 1930; pl. 2., Fig. 2); MMUL 1447/G 12931, an epiplastron (Liebus, 1930; pl. 2., Fig. 3); NMP P/9640(4)/Pv 12303, an epiplastron (Liebus, 1930; pl. 2., Fig. 4); MMUL I/G 1486/G 12939, a hyoplastron (Liebus, 1930; pl. 2., Fig. 5); MMUL 1035/G 12915, a right hypoplastron (Liebus, 1930; pl. 2., Fig. 6); MMUL 1038/G 12918, a xiphiplastron (Liebus, 1930; pl. 3., Fig. 1); MMUL 1036/G 12916, a pelvic girdle (Liebus, 1930; pl. 3., Fig. 3); MMUL 1041/G 12921, an imprint of MMUL 1036/G 12916; MMUL 1442/G 10193, a complete skull (Liebus, 1930; pl. 3., Fig. 3); MMUL 1443/G 12926, an imprint of MMUL 1442/G 10193; MMUL 631/G 12912, a partial pectoral girdle (Liebus, 1930; pl. 3., Fig. 4); MMUL 1451/G 12933, a cervical vertebra (Liebus, 1930; pl. 3., Fig. 5); MMUL 1452/G 12934, a cervical vertebra; MMUL 1461/G 12937, a coracoid; MMUL 1037/G 12917, a skull with a xiphiplastron; MMUL 1048/G 10194, a skull with preserved palatal area; MMUL 1042/G 12922, an imprint of MMUL 1037/G 12917; MMUL 1040/G 12919, a skull; MMUL 1045/G 12925, a mandible; MMUL 1450/G 12932, a radius and ulna; MMUL 1453/G 12935, a fibula; MMUL 1446/G 12930, a tibia with a fragment of cervical vertebra; MMUL 1445/G 12928, an imprint of MMUL 1446/G 12930 together with epiplastral distal fragment; MMUL 1462/G 12938, a left femur. Compared to Georgalis & Joyce (2017), we included historic and current valid numbers to avoid confusion, as well as improved the numbering of material published by Liebus (1930). MMUL 633/2a/G 12908 is correctly MMUL 633/2ab/G 12908/G 12941 and MMUL 633/2b/G 12941 is MMUL 633/1/G 12910 (Liebus, 1930; pl. 1., Figs. 1 and 2). MMUL 1486/G 12939 is correctly MMUL I/G 1486/G 12939 (Liebus, 1930; pl. 2., Fig. 5). A skull MMUL 1043/G 12919 is correctly MMUL 1040/G 12919. MMUL 1443/G 12926 is an imprint of MMUL 1442/G 10193. MMUL 1461/G 12937 is a coracoid, instead of an epiplastron. MMUL 1446/G 12930 (not MMUL 1446/G 12929) refers to a tibia with a fragment of cervical vertebra. MMUL 1445/G 12928 (not MMUL 1445/G 12930) refers to its imprint plus an epiplastral distal fragment. Moreover, we added MMUL 1452/G 12934, a cervical vertebra, surprisingly not mentioned by Liebus (1930), but belongs to the same collection. Other material was not classified as syntypes as they were not included in the thesis made by Liebus (1930). The material housed in Teplice (RMT PA 1309, RMT PA 1310, RMT PA 1683, RMT PA 1684, RMT PA 1686, and RMT PA 1693) came into the museum between 1898 –1910, which means that Liebus probably checked them, but only described RMT PA 1308/1 and RMT PA 1308/2 as *T*. *aspidiformis* (Liebus, 1930, pl. 4.). MMG CST 621 was also considered here as a cervical vertebra of the fossil alligatoroid *Diplocynodon*, as Laube (1901) did. Although NMP Pv 11668 does not have a donor record nor year of donation, its lithology confirms that the specimen comes from Břešt’any. We considered NMP Pv 11668 as referred in the Table 1.

**Figure 8:**
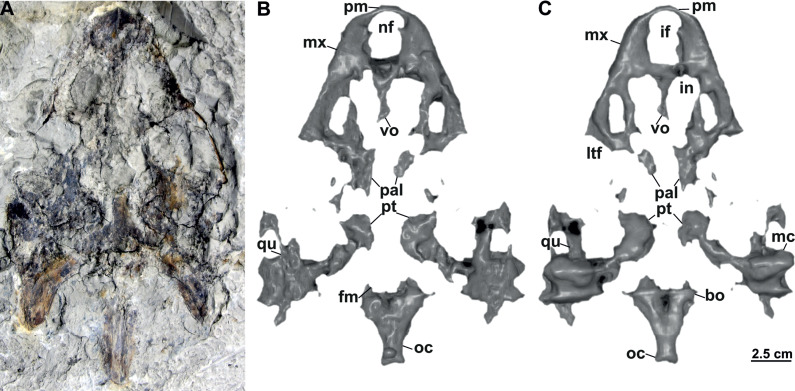
Skull and schematic drawings of *Rafetusbohemicus* from Břešt’any. Partial skull and digital rendering derived from CT data of NMP Pv 11668 in dorsal (A–B) and ventral (C) views. Abbreviations: bo, basioccipital; fm, foramen magnum; if, intermaxillary foramen; in, internal naris; ltf, lower temporal fossa; mc, mandibular condyle; mx, maxilla; nf, nasal fossa; oc, occipital condyle; pal, palatine; pm, premaxilla; pt, pterygoid; qu, quadrate; vo, vomer.

**Type locality.** Břešt’any (Preschen), Czechia.

**Differential diagnosis:** *Rafetus bohemicus* differs from *Trionyx vindobonensis* (Peters, 1855) by a shorter and broader snout, a non-concave medial edge of the maxilla in palatal view, a short intermaxillary suture developed between the intermaxillary foramen and the internal naris, and a large internal naris. Compared to extant species *Rafetus euphraticus* (Daudin, 1801) and *Rafetus swinhoei* (Gray, 1873), the basisphenoid is in contact with vomer but not with the palatines. Together with *R. euphraticus*, *R. bohemicus* has a median contact of the maxillae, which separates the anterior from the posterior portions of the vomer. Pectoral girdle has a very short ventromedial process (acromion process), and a single anterolateral hyoplastral process that is not divided into multiple processes. Compared to *R. euphraticus*, *R. bohemicus* has a more pronounced mediolateral constriction of the xiphiplastral elements. Also, *R. bohemicus* is smaller compared to extant *R. swinhoei* (Georgalis & Joyce, 2017).

### Preservation

All skulls are preserved in fine-grained limnic clay.

**Figure 9:**
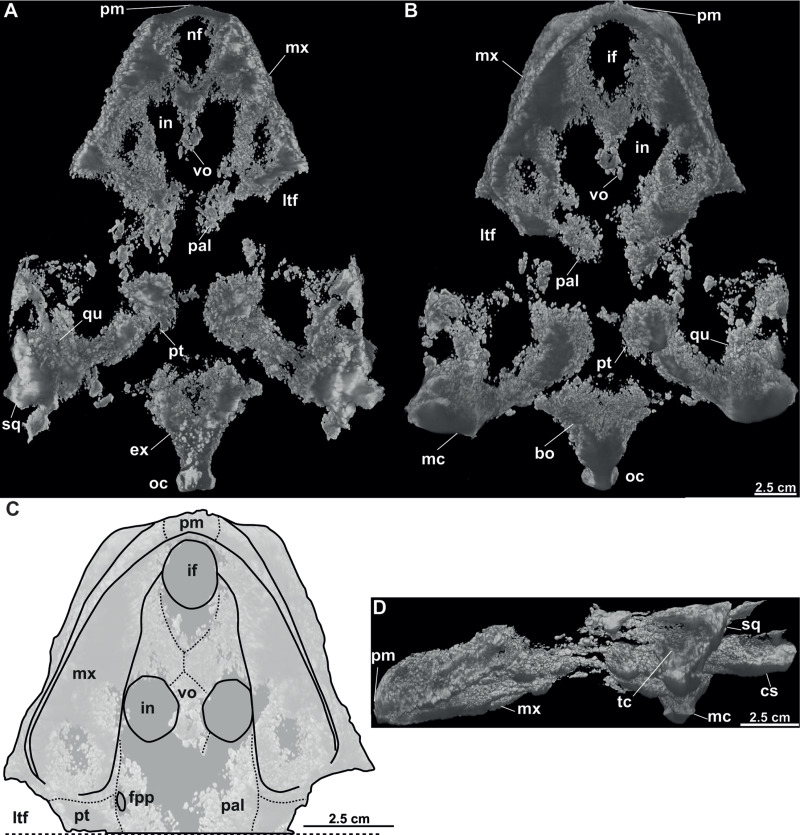
CT model and schematic drawing of *Rafetusbohemicus* from Břešt’any. Digital rendering derived from CT data of NMP Pv 11668 in dorsal (A), ventral (B–C) and left lateral (D) views. Abbreviations: bo, basioccipital; cs, crista supraoccipitalis; ex, exoccipital; fpp, foramen palatinum posterius; if, intermaxillary foramen; in, internal naris; ltf, lower temporal fossa; mc, mandibular condyle; mx, maxilla; nf, nasal fossa; oc, occipital condyle; pal, palatine; pm, premaxilla; pt, pterygoid; qu, quadrate; sq, squamate; tc, tympanic cavity; vo, vomer.

**NMP Pv 11668**: The specimen is embedded in a slab and covered by a thin layer of clay (Fig. 8A). The left temporal arch and some bones of the skull roof are missing. Also, the external naris together with the posterior part of the skull is partially preserved (Figs. 8A–8C, 9A).

**Figure 10:**
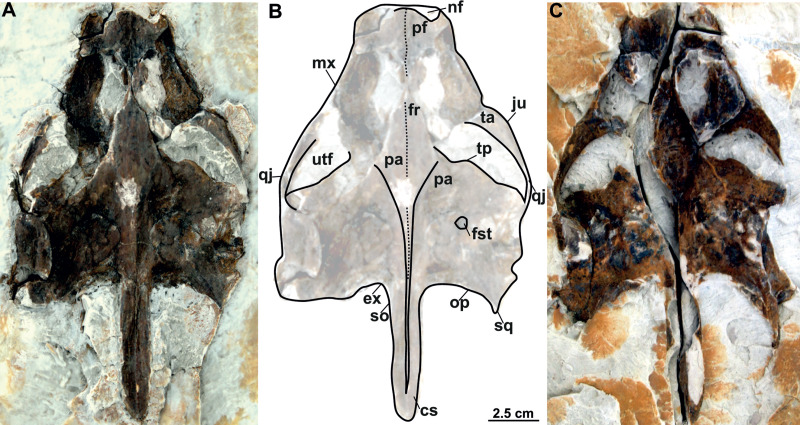
Skull and schematic drawing of *Rafetus bohemicus* from Břešt’any. Original (MMUL 1442/G 10193, syntype) and its imprint (MMUL 1443/G 12926, syntype) of a partial skull in dorsal (A–C) and visceral (C) views. Abbreviations: cs, crista supraoccipitalis; ex, exoccipital; fr, frontal; fst, foramen stapedio-temporale; ju, jugal; mx, maxilla; nf, nasal fossa; op, opisthotic; pa, parietal; pf, prefrontal; qj, quadratojugal; so, supraoccipital; sq, squamosal; ta, temporal arch; tp, trochlear process; utf, upper temporal fossa.

**MMUL 1442/G 10193 and MMUL 1443/G 12926 (syntypes)**: MMUL 1442/G 10193 preserves the skull roof, the occipital area and partially the snout (Figs. 10A–10C). Both squamosal posterior processes are eroded. Its imprint (MMUL 1443/G 12926) is cracked in anteroposterior direction (Fig. 10C).

**Figure 11:**
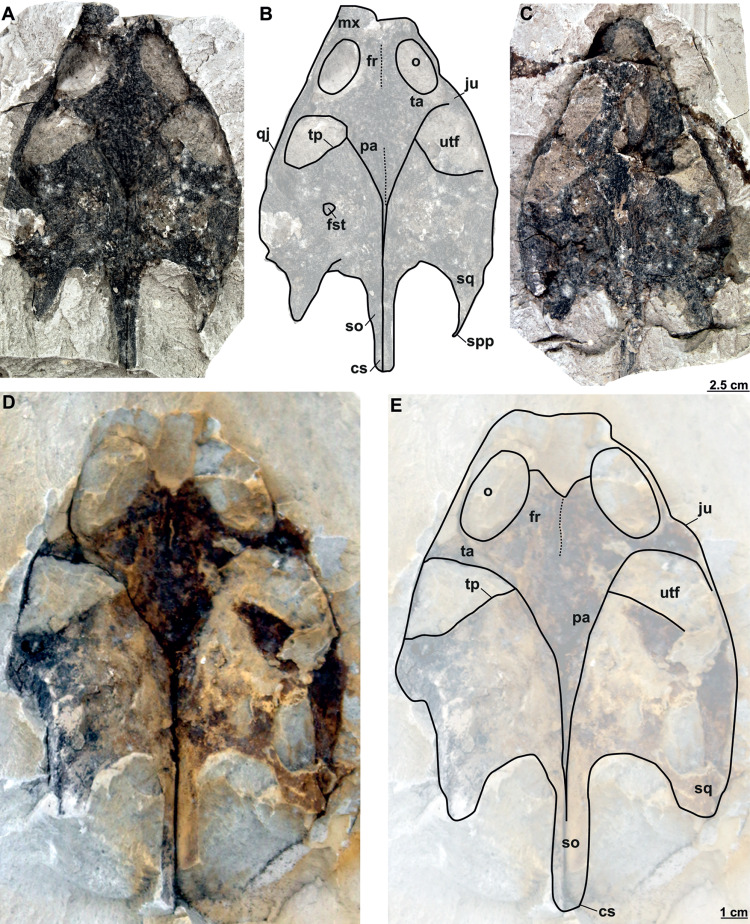
Skulls and schematic drawings of *Rafetusbohemicus* from Břešt’any. Original (RMT PA 1309) and its imprint (RMT PA 1310) of a partial skull in dorsal (A–B) and visceral (C) views. Partial skull MMUL 1049/G 10196 (syntype) in dorsal (D–E) views. Abbreviations: cs, crista supraoccipitalis; fr, frontal; fst, foramen stapedio-temporale; ju, jugal; mx, maxilla; o, orbit; pa, parietal; qj, quadratojugal; so, supraoccipital; spp, squamosal posterior processes; sq, squamosal; ta, temporal arch; tp, trochlear process; utf, upper temporal fossa.

**RMT PA 1309 and RMT PA 1310**: RMT PA 1310 is a partial complete skull, where the most anterior part of the snout is eroded and both squamosal processes are damaged (Figs. 11A–11B). RMT PA 1309 is the imprint of the skull, which is poorly preserved (Fig. 11C).

**MMUL 1049/G 10196 (syntype)**: It is a negative imprint of the skull lacking the snout and both posterior squamosal processes (Figs. 11D–11E).

**Figure 12:**
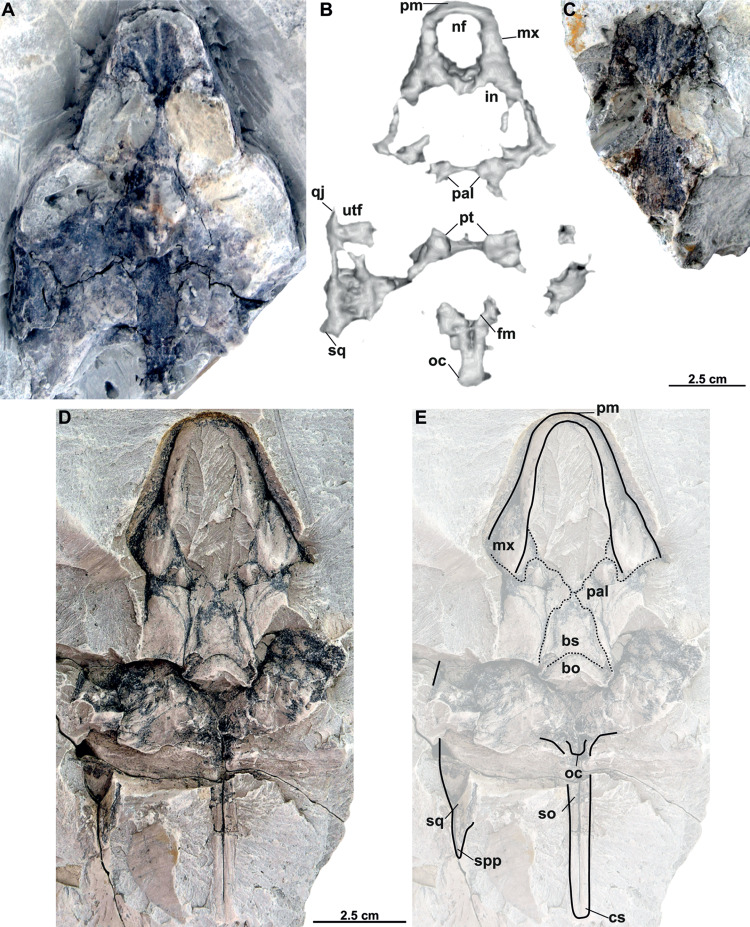
Skulls and schematic drawings of *Rafetusbohemicus* from Břešt’any. Original (MMUL 1037/G 12917, syntype) and its imprint (MMUL 1042/G 12922, syntype) of a partial skull and its digital rendering derived from CT data in dorsal (A–B) and visceral (C) views. Partial skull MMUL 1048/G 10194 (syntype) in ventral (D–E) views. Abbreviations: bo, basioccipital; bs, basisphenoid; cs, crista supraoccipitalis; fm, foramen magnum; in, internal naris; mx, maxilla; nf, nasal fossa; occipital condyle; pal, palatine; pm, premaxilla; pt, pterygoid; qj, quadratojugal; so, supraoccipital; spp, squamosal posterior processes; sq, squamosal; utf, upper temporal fossa.

**MMUL 1037/G 12917 and MMUL 1042/G 12922 (syntypes)**: MMUL 1037/G 12917 is an almost complete skull (Figs. 12A–12B), whereas MMUL 1042/G 12922 represents its imprint (Fig. 12C). The skull roof and both temporal arches are eroded. However, MMUL 1037/G 12917 preserves the orbits, prefrontals and frontals (Fig. 12A).

**MMUL 1048/G 10194 (syntype)**: It is an imprint of the partial skull preserved in ventral view, which is formed by three parts glued together (Figs. 12D–12E). The outline of the snout is well preserved and the sutures of some palatal bones (*i.e.,* the basisphenoid, basioccipital, maxillae and pterygoids) are visible (Fig. 12D).

### Description

Six partial skulls are available from Břešt’any: NMP Pv 11668 (Figs. 8A–8C, 9A–9D); MMUL 1442/G 10193 and MMUL 1443/G 12926 (syntypes, Figs. 10A–10C); RMT PA 1310 and RMT PA 1309 (Figs. 11A–11C); MMUL 1049/G 10196 (syntype, Figs. 11D–11E); MMUL 1037/G 12917 and MMUL 1042/G 12922 (syntypes, Figs. 12A–12C); and MMUL 1048/G 10194 (syntype, Figs. 12D–12E). Of these six skulls, the three most complete (Figs. 8A–8C, 9A–9D, 11A–11C, 12A–12C) were scanned. All measurements should be treated as estimates because the posterior end of the supraoccipital crest is not preserved perfectly in any of the skulls. RMT PA 1310 is the largest skull, with an approximate length of 19.2 cm from the anterior rim of the orbit to the posterior end of the supraoccipital crest. The same distance is available for the following specimens: 14.8 cm for NMP Pv 11668, 13.7 cm for MMUL 1442/G 10193, and 9.8 cm for MMUL 1049/G 10196. This notable difference in size between skulls might be due to different ontogenetic stages or to intraspecific variation. Though the morphology of all skulls is very similar, larger individuals show a more developed supraoccipital crest and squamosal posterior process. Liebus (1930) could only interpret some dorsal parts of the syntype skull MMUL 1442/G 10193 (Figs. 10A–10C); however, the new specimens clearly reveal most of the ventral side, as well as the missing parts of the dorsal roof. Overall, the skulls are triangular or pear-shaped, longer than wide, and with a short and broad snout (Figs. 8A–8C, 9A–9C, 12A–12C, 12D–12E). The orbits are dorsally oriented, and the skull roof is reduced by a deep temporal emargination.

**Premaxilla**. The virtual 3D model of NMP Pv 11668 allows to evaluate the premaxilla (Figs. 8B–8C, 9A–9C): small and fused with its counterpart, forming the anterior tip of the snout. The premaxillae seem excluded from the external naris, as in all trionychids (Meylan, 1987). The premaxilla, together with the maxilla, make up the triturating surface. In dorsal view, these bones are partly visible as they are located just below the prefrontals (*e.g.*, Figs. 10A–10C). Given that the maxilla contacts with its counterpart anteromedially along a medial short intermaxillary suture, the premaxillae seem to be in contact with the intermaxillary foramen.

**Maxilla**. The maxilla is a large and flat bone that forms most of the palate area. It is partially preserved in all specimens. It contacts the premaxilla anteromedially, the vomer medially (surrounding the intermaxillary foramen), the palatine also medially (surrounding the internal naris), the pterygoid posteriorly and the prefrontal dorsomedially (forming the ventral rim of the orbit). On the triturating surface of MMUL 1048/G 10194 (Figs. 12D–12E), two different ridges can be recognized: the labial ridges develop longitudinally and form the lateral borders of the triturating surface; and the lingual ridges, which are slightly crenulated and almost straight (non-convex; Figs. 9B–9C, 12D–12E). The palatal area, limited by the lingual ridges, is markedly concave (Figs. 9B–9C). As observed from NMP Pv 11668, a medial short intermaxillary suture separates the vomer (Figs. 9B–9C). The internal nares are large and rounded, and similar in size to the intermaxillary foramen.

**Prefrontal**. The prefrontal is fully preserved in MMUL 1442/G 10193 (Figs. 10A–10C) and MMUL 1037/G 12917 (Figs. 12A–12C). This bone is rectangular and longer than wide: it contacts the maxilla laterally, the frontal posteriorly, and abuts its counterpart along a straight median suture. The prefrontal contributes to the dorsal margin of the external naris and the orbital margin posterolaterally.

**Frontal**. The frontal is a trapezoidal, paired bone, and is preserved in four of the skulls (Figs. 10A–10C, 11A–11E, 12A–12C). This element contacts the prefrontal anteriorly, the postorbital laterally, the parietal posteriorly and its counterpart medially. The frontals form a substantial portion of the dorsomedial orbital margin.

**Postorbital**. The postorbital is the smallest roofing bone, only available in two specimens (Figs. 11A–11B, 11D–11E). It contacts the frontal anteromedially, the jugal laterally, and the parietal posteromedially. It forms the posterior rim of the orbit; however, it is not clear whether it contributes to the rim of the upper temporal emargination.

**Jugal**. The jugal is partially preserved in three specimens, but only available in dorsal view (Figs. 10A–10C, 11A–11E). On the dorsal skull roof, the jugal contributes to the posterior orbital margin. It contacts the maxilla anteriorly (just behind the orbit), the postorbital medially (forming the temporal arch), and the quadratojugal posterolaterally.

**Parietal**. The parietal is a triangular elongate paired bone, preserved in three skulls (Figs. 10A–10C, 11A–11E). Although it makes up a large part of the skull roof, it is medially reduced by the upper temporal emargination. On the skull roof, the parietal contacts the frontal anteriorly, the postorbital and jugal anterolaterally (*i.e.,* the medial edge of the upper temporal fossa), the prootic laterally, its counterpart medially, and the supraoccipital posteriorly. The trochlear process is fully exposed dorsally and seems to be formed by the parietal and prootic (Figs. 10A–10B, 11A–11B, 11D–11E). The parietal does not seem to contact the squamosal at any point.

**Quadratojugal**. The quadratojugal is poorly preserved in all specimens. In the dorsal view, the quadratojugal is a small and elongated bone that is reduced to a bar and restricted to the temporal arch; it contacts the jugal anteriorly (forming the posteromedial part of the temporal arch), the quadrate posteromedially and the squamosal posteriorly (Figs. 10A–10B, 11A–11E).

**Squamosal**. The squamosal is available in all specimens, but the best-preserved ones are those of MMUL 1442/G 10193 (Figs. 10A–10C) and RMT PA 1310 (Figs. 11A–11B). The squamosal contacts the quadratojugal anteriorly, the quadrate medially and the opisthotic posteromedially. The squamosal forms the dorsal rim of the tympanic cavity and the posterolateral corners of the skull. As is seen in RMT PA 1309–10 (Figs. 11A–11B) and MMUL 1048/G 10194 (Figs. 12D–12E), the squamosal posterior processes are well developed posteriorly.

**Vomer**. It is an elongated bone only well preserved in NMP Pv 11668 (Figs. 9B–9C). In ventral view, the maxillae do not prevent the vomer from contributing to the posterior edge of the intermaxillary foramen (Figs. 9B–9C, 15A). This bone not only splits the palatines from each other medially, but further its posterior tip is in touch with the basisphenoid, and consequently, separates the pterygoids along the midline (Figs. 12D–12E). The preserved portion of the vomer broadly contributes to the medial rim of the internal nares, which are large and suboval.

**Palatine**. The palatine is an elongated paired element that can only be observed in NMP Pv 11668 (Figs. 8A–8C, 9A–9C). This bone contacts the maxilla anterolaterally, the vomer medially forming most of the posterior rim of the internal nares, and the pterygoid posteriorly. The right foramen palatinum posterius is preserved and located close to the palatine-pterygoid-maxilla suture: it is small and not divided into multiple openings (Figs. 9B–9C).

**Pterygoid**. The pterygoid is a large, paired bone partially available in NMP Pv 11668 and MMUL 1048/G 10194 (Figs. 8A–8C, 9A–9C). It has a broad anterior process that forms a transverse contact with the maxilla, just posterior to the triturating surface. It also contacts the palatine and vomer anteromedially, the basisphenoid medially, the basioccipital posteromedially and the quadrate posterolaterally. This bone broadly contributes the lower temporal fossa.

**Quadrate**. The quadrate is rather poorly preserved in all specimens. In the lateral view, the tympanic cavity seems completely enclosed by the quadrate (Fig. 9D). This element is in contact with the prootic anteriorly, the squamosal laterally (at least in dorsal view), the opisthotic posteriorly, and the pterygoid ventromedially. Although the CT model of NMP Pv 11668 shows much of the otic chamber, the columella auris is not preserved. A slightly robust and low mandibular condyle is clearly visible in NMP Pv 11668 (Figs. 9B, 9D), where the lateral facet is larger than the medial one. The foramen stapedio-temporale is preserved, although it is impossible to evaluate which bones are involved (Figs. 10A–10B, 11A–11B).

**Prootic**. The prootic is broadly exposed in dorsal view within the upper temporal fossa (Figs. 10A–10C, 11A–11B, 11D–11E). It is longer than wide and participates in the foramen stapedio-temporale according to the CT model of NMP Pv 11668. The prootic seems overlapped by the squamosal posterolaterally and the parietals medially. It also contacts the supraoccipital posteromedially, the opisthotic posteriorly and the pterygoid ventromedially.

**Opisthotic**. The opisthotic is partially preserved in all the specimens, except for MMUL G 10194, in which it is completely missing. It seems longer than wide and forms the posterior part of the skull between the squamosal and supraoccipital. Within the upper temporal fossa, the opisthotic contacts the prootic anteriorly, the supraoccipital anteromedially, the quadrate anterolaterally, and the exoccipital posteromedially (Figs. 10A–10C, 11A–11B, 11D–11E). In the opisthotics preserved in MMUL 1442/G 10193, RMT PA 1310 and MMUL 1049/G 10196, this bone is not directly involved in the foramen stapedio-temporale.

**Basisphenoid**. The basisphenoid is an elongate subtriangular bone that is preserved only in MMUL 1048/G 10194 (Figs. 12D–12E). It contacts the vomer anteromedially, both pterygoids laterally, and the basioccipital posteriorly.

**Basioccipital**. The basioccipital is well preserved in MMUL 1048/G 10194 (Figs. 12D–12E). In ventral view, this subsquare bone is located posterior to the basisphenoid and contacts the pterygoids laterally and the exoccipitals posterodorsally. The basioccipital contributes to the ventral third of the occipital condyle.

**Exoccipital**. The exoccipital is a paired element that surrounds the lateral rim of the foramen magnum and forms the two dorsal thirds of the occipital condyle. It is partially preserved in all specimens, except for MMUL 1048/G 10194, in which it is eroded (Figs. 12D–12E). It contacts the opisthotic anteriorly, the supraoccipital dorsomedially, and the basioccipital ventromedially.

**Supraoccipital**. The supraoccipital is relatively well preserved in all the available skulls (Figs. 8A, 10A–10C, 11A–11B, 11D–11E, 12D–12E). Ventrally, it broadly contacts the exoccipital and opisthotic, and forms the dorsal margin of the foramen magnum (Figs. 11B–11C, 12B). As can be discerned in dorsal view, the supraoccipital contacts the parietal anterodorsally, the prootic anterolaterally, and the opisthotic laterally. The most posterior element of the skull (*i.e.,* the supraoccipital crest) is complete in MMUL 1442/G 10193 and MMUL 1049/G 10196; it is elongated, inverted T-shaped in cross section, and extends posteriorly further than the squamosal processes (Figs. 10A–10C, 11A–11E, 12D–12E).

**Cranial fenestrae and openings.** The external naris is encircled by the prefrontals and maxillae (Figs. 8B–8C, 10B and 12B). In the best-preserved skull (MMUL 1442/G 10193), it is relatively large and opens anteriorly. The orbit is delimited by the maxilla, prefrontal, frontal, postorbital and jugal, as it can be discerned in RMT PA 1310 and MMUL 1049/G 10196 (Figs. 11A–11B, 11D–11E). It is elliptical and slightly larger than the external naris. The upper temporal fossa is wider but anteroposteriorly shorter than the orbit, being separated from the latter by a vaguely robust temporal arch (Figs. 11A–11B, 11D–11E). Although it is difficult to evaluate the temporal emargination, it seems to be delimited anteriorly by the postorbital and jugal, the squamosal laterally, and the parietal medially (Figs. 10B, 11B, 11E). The lower temporal fossa is not sufficiently preserved in any skull: in NMP Pv 11668 it is partly covered by matrix (Fig. 8A), whereas in MMUL 1048/G 10194 it is eroded (Figs. 12D–12E). Thus, its original shape cannot be assessed with confidence. The foramen magnum is made up by the exoccipital and supraoccipital. It is oval-shaped and is much larger than the occipital condyle (Figs. 8B–8C), being delimited by the supraoccipital and exoccipital. The tympanic cavity is poorly preserved in all skulls, except for NMP Pv 11668 (Fig. 9D), where it appears as wide as high and completely enclosed by the quadrate.

### Carapace

**Figure 13:**
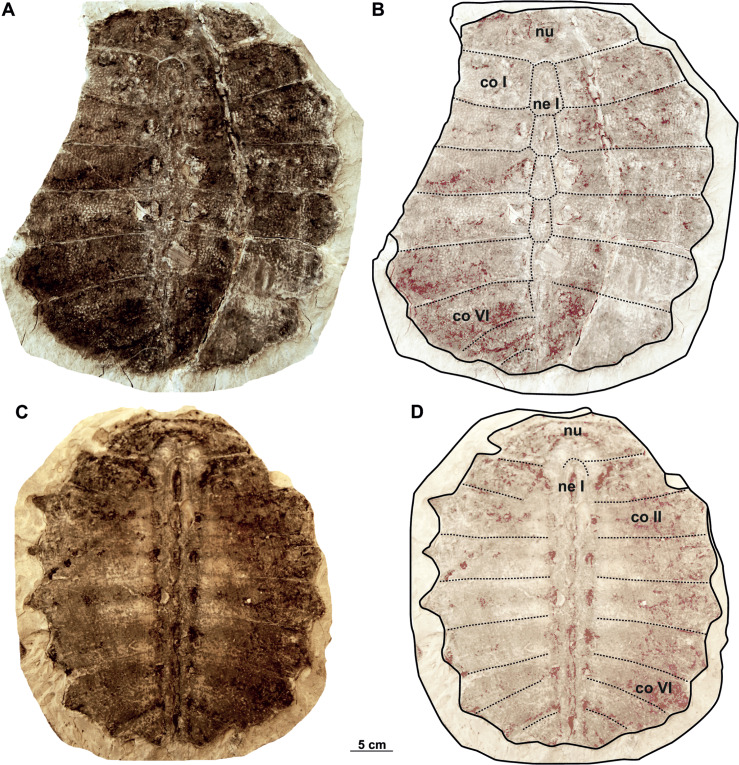
Syntype carapaces of *Rafetus bohemicus* from Břešt’any described by Liebus (1930). The original carapace MMUL 633/2ab/G 12908/G 12941 in dorsal (A–B) view. Its imprint MMUL 633/1/G 12910 in visceral (C–D) view. Abbreviations: co I, costal I; co II, costal II; co VI, costal VI; ne I, neural I; nu, nuchal.

**Figure 14:**
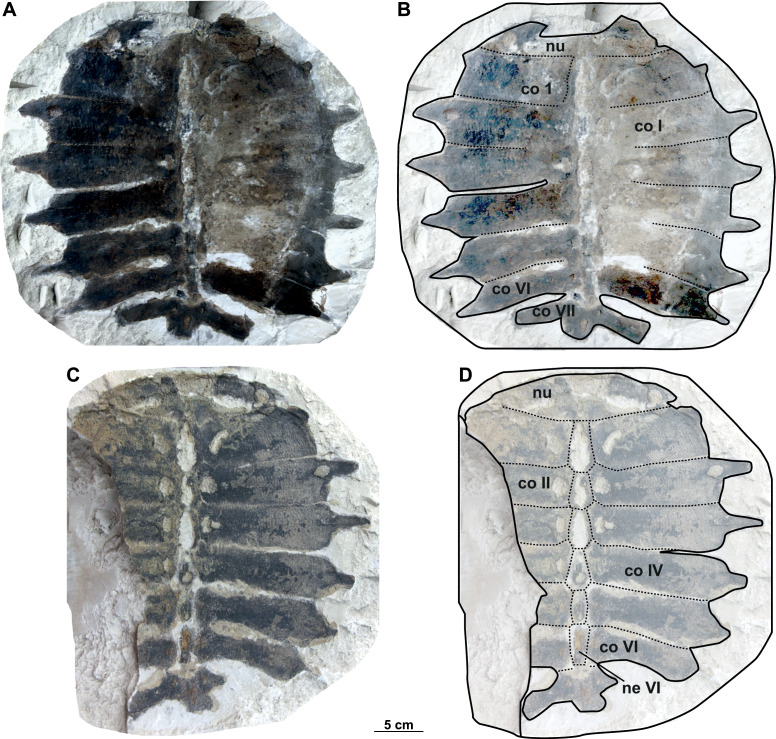
Syntype carapaces of *Rafetus bohemicus* from Břešt’any described by Liebus (1930). The original of the carapace NMP 1485a/Pb 2a in dorsal (A–B) views. Imprint NMP 1485b/Pb 2b in visceral (C–D) views. Abbreviations: co II, costal II; co IV, costal IV; co VI, costal VI; co VII, costal VII; ne VI, neural VI; nu, nuchal.

The two carapaces with its imprints (syntypes) of *Rafetus bohemicus* described by Liebus (1930) are available: MMUL 633/1/G 12910 (Figs. 13C–13D) is almost complete and well preserved, whereas MMUL 633/2ab/G 12908/G 12941 (Figs. 13A–13B, original of MMUL 633/1/G 12910) and NMP 1485a–b/Pb 2a–b (Figs. 14A–14D, original and imprint respectively) lack the left lateral portions of costals. However, in none of these specimens the sutures of neural VII and costal VIII are discerned. The carapaces belong to subadults and are roughly similar in size, *i.e.,* the midline length is approximately 30 cm, they appear notably thin; however, their thickness cannot be fully measured as they remain embedded in slabs. The pygal, suprapygal and peripheral plates are absent. MMUL 633/2ab/G 12908/G 12941, MMUL 633/1/G 12910 and NMP 1485a–b/Pb 2a–b greatly resemble the holotypes of *T. pontanus* and *T. preschenensis*, in which the carapace is suboval in shape and slightly longer than wide. The lateral borders have the rib extensions without callosity, which reach up to 2.5 cm beyond the carapacial border (Figs. 14A–14D). The sculpturing pattern of MMUL 633/2ab/G 12908/G 12941 and NMP 1485a–b/Pb 2a–b is quite comparable to all previous carapaces described from Břešt’any in having fine ridges and round to angular pits that form an irregular pattern throughout the surface of the carapace (Figs. 13A–13B, 14C–14D). This surface sculpturing extends anteroposteriorly across the costal plates but never appears to branch, and seems to be more subtle in the neural series (Figs. 13A–13B). No specimens have scutes on the carapace.

**Nuchal**. It is the most anterior element of the carapace and is preserved in all three carapaces. This plate is approximately three times wider than long and the costiform processes is partially preserved in MMUL 633/1/G 12910 (Figs. 13C–13D) and NMP 1485a/Pb 2a–b (Figs. 13A–13B, 14A–14B). As in other adult specimens from Břešt’any, the anterior edge of the nuchal is slightly concave medially (Figs. 13C–13D). Viscerally, the first thoracic vertebra is situated at the posterior part of the nuchal plate (Figs. 13C–13D). The preneural plate and suprascapular fontanelles are lacking.

**Neurals**. The neural series is formed by seven plates and is almost complete in two specimens (MMUL 633/2ab/G 12908/G 12941 and NMP 1485a/Pb 2a–b; Figs. 13A–13B, 14C–14D). Neural I, which is the largest plate, is hexagonal with short sides behind and contacts the costals I–II laterally. Neurals II–IV are also hexagonal but decrease in size posteriorly. Neural V is a subrectangular and narrow element that prevents costals V from contacting one another along the midline, marks the reversal in the arrangement of neurals. As for the last elements, neural VI is hexagonal with shorter anterior sides, whereas it is not possible to evaluate the shape of neural VII as its sutures are obliterated in all three carapaces.

**Costals**. The carapace includes eight costals, which are paired elements, well developed laterally. Costals I–II are slightly trapezoidal and oriented anteriorly; it is noteworthy that both the anterior and posterior sutures of costal I are transverse (MMUL 633/2ab/G 12908/G 12941 and NMP 1485a/Pb 2a–b; Figs. 13A–13B, 14A–14B). Costal III is rectangular (*i.e.,* it has similar dimensions proximally and distally), oriented laterally, and displays straight anterior and posterior sutures (Figs. 13B, 13D). Costals IV–VII are trapezoidal, oriented posteriorly, and tend to be expanded distally. Costal VIII is very reduced, ranging from subtriangular to trapezoidal in shape. Although all costals have similar anteroposterior dimensions along the midline, costal I is slightly more elongated. As can be observed in NMP 1485a/Pb 2a, the lateral extensions (*i.e.,* ribs) are preserved only on costals I–VI.

### Plastron

**Figure 15:**
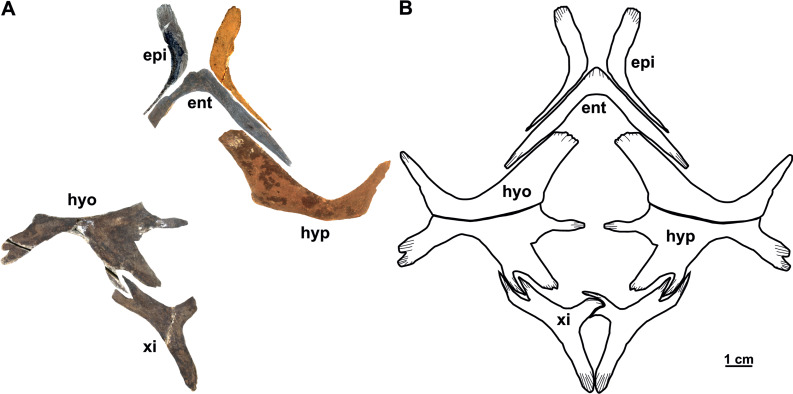
The plastron (syntype) of *Rafetus bohemicus* from Břešt’any. Plastron elements in ventral (A) view; left (NMP P/9640(4)/Pv 12303) and right (MMUL 1447/G 12931) epiplastron; entoplastron (MMUL 1444/G 12927); right hyoplastron (MMUL I/G 1486/G 12939); left hypoplastron (MMUL 1035/G 12915); left xiphiplastron (MMUL 1038/G 12918). A schematic drawing of the plastron in ventral (B) view. Abbreviations: ent, entoplastron; epi, epiplastron; hyo, hyoplastron; hyp, hypoplastron; xi, xiphiplastron.

The plastral material (syntypes) consists of two complete (right and left) epiplastra, an almost complete entoplastron, a right hyoplastron, a left hypoplastron, and an almost complete left xiphiplastron (Figs. 15A–15B). As in all trionychids, the plastral elements are quite reduced. The visceral surface of the plastron is smooth, whereas in ventral view only the hyoplastra and hypoplastra are associated with callosities: they are poorly developed and mainly restricted along the hyo-hypoplastral suture. The sculpturing pattern of the plastral callosities are irregularly punctate (Fig. 15A).

**Epiplastron**. The epiplastron is the anterior most paired bone of the plastron. NMP P/9640(4)/Pv 12303 (left portion) and MMUL 1447/G 12931 (right portion) are the two available elements. It is hockey stick-shaped and lacks callosities, both on ventral and visceral sides. The anterior process is rounded and flat, whereas the lateral one taper distally and terminate in an acute process (Figs. 15A–15B).

**Entoplastron**. This plastral element is located in the anteromedial part of the plastron: it is inverted V-shaped and lacks any evidence of callosities (Figs. 15A–15B). The isolated entoplastron MMUL 1444/G 12927 has completely preserved the right posterolateral process and the central plateau, which is transverse. The angle between the lateral processes is about 90°. The entoplastron is not fused to the hyoplastron.

**Hyo/Hypoplastron**. Both bones are the largest elements of the plastron and comprise the latter’s central portion. The right hyoplastron (MMUL I/G 1486//G 12939) is slender, wider than long, and reduced anteroposteriorly. It has a single lateral process that is developed in anterolateral direction (∼45°). Although the distal tip of the anteromedial process is missing, it does not seem to be divided into multiple processes (*i.e.,* fingering). The posterior margin of this element is concave and the contact with the hypoplastron was osseous. The hypoplastron (MMUL 1035/G 12915; Figs. 15A–15B) is wider than long and has one pointed hypoplastral medial process that develops perpendicular to the axis plane. The lateral hypoplastral process is double and runs in posterolateral direction. Similarly, the posterior hypoplastral process is double, but the most medial one is larger and slightly bilobed (Figs. 15A–15B). Both processes form a deep triangular notch for ligamentous articulation with the xiphiplastron. Given that this element is not available in ventral view, it is not possible to evaluate its callosities.

**Xiphiplastron**. One almost complete left (MMUL 1038/G 12918; Figs. 15A–15B) and right xiphiplastron (MMUL 1037/G 12917; including its imprint MMUL 1042/G 12922) are available. This element forms two anterior processes, of which the lateral one is double and fits the posterolateral processes of the hypoplastron (Figs. 15A–15B). Although its distal portion is missing, the medial process appears to be narrow. The xiphiplastron, furthermore, forms an elongated posterior process, which is almost twice longer than in extant *Rafetus* species. The ventral surface of this element has no callosity.

### Vertebrae and appendicular elements

**Figure 16:**
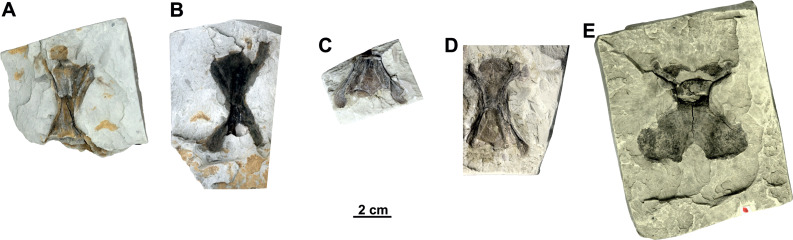
Isolated cervical vertebrae of *Rafetus bohemicus* from Břešt’any. Cervical vertebra III MMUL 1452/G 12934 (syntype) in ventral (A) view. Cervical vertebra IV MMUL 1451/G 12933 (syntype) in ventral (B) view. Cervical vertebra IV NMP Pv 12302 (syntype) in ventral (C) view. Cervical vertebra VI NMP Pb 51 (syntype) in ventral (D) view. Cervical vertebra VIII MMG CST 621 in dorsal (E) view.

**Figure 17:**
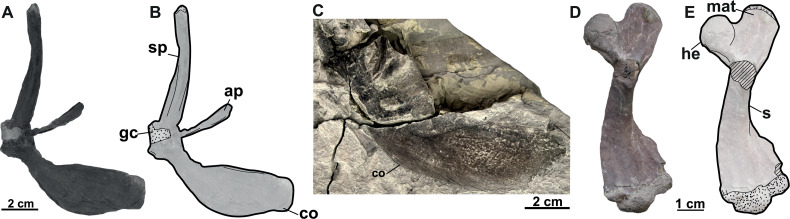
The forelimbs of *Rafetus bohemicus* from Břešt’any. Right pectoral girdle MMUL 631/G 12912 (syntype) in anteroventral (A–B) view. Right coracoid MMUL 1461/G 12937 (syntype) in anteroventral (C) view. Right humerus RMT PA 1684 in anteroventral (D–E) view. Abbreviations: ap, acromion process; co, coracoid; gc, glenoid cavity; he, humeral epiphysis; mat, major trochanter; s, shaft; sp, scapular process.

**Figure 18:**
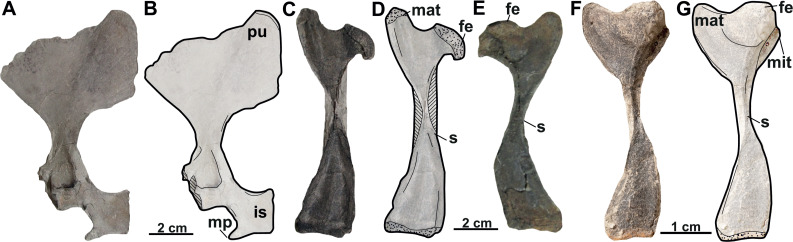
The hind limbs of *Rafetus bohemicus* from Břešt’any. Left pubis and ischium RMT PA 1686 in dorsal (A–B) views. Right femur RMT PA 1683 in posterior (C–D) views. Left femur RMT PA 1693 in posterior (E) view. Right femur RMT PA 1308/1 in dorsal (F–G) views. Abbreviations: fe, femoral epiphysis; is, ischium; mat, major trochanter; mit, minor trochanter; mp, metischial process; pu, pubis; s, shaft.

The Břešt’any site yielded a small amount of disarticulated material pertaining to the cervical vertebrae (Figs. 16A–16E), pectoral (Figs. 17A–17C) and pelvic (Figs. 18A–18B) girdles, and limbs (humerus, Figs. 17D–17E; femur, Figs. 18C–18G).

**Cervical vertebrae**. Five isolated cervical vertebrae were recovered (Figs. 16A–16E). All these cervical vertebrae are opisthocoelous with anterior condyle, with the exception of cervical VIII, originally described as *Diplocynodon* sp. (Laube, 1901; pl. 7., Fig. 7).

The cervical III (MMUL 1452/G 12934, syntype; Fig. 16A) is long, almost two times longer than wide, and has a narrow and large anterior condyle. The dorsal surface of the neural arch is partially preserved and only the right prezygapophysis can be discerned. Similarly, the posterior half of the vertebra with postzygapophyses is missing. A short mid-ventral is preserved.

As for cervical IV, two elements are available: MMUL 1451/G 12933 (syntype) and NMP Pv 12302 (Figs. 16B–16C, respectively). As seen in MMUL 1451/G 12933, the neural body contains a crack and whole vertebra is dorsoventrally flattened together: both pre- and postzygapophyses are preserved, whereas the right prezygapophysis is missing. This element is clearly slender despite that the most distal end of the left postzygapophyses is eroded (*i.e.,* the facet). As whole posterior part of NMP Pv 12302 is missing, only prezygapophyses are evaluable, which are slender and directed backward.

Unlike previous vertebrae, NMP Pb 51 (Fig. 16D) is more robust, shorter, and contains a small concavity in the most posteromedial tip. Both prezygapophyses are preserved but only represented by their proximal parts. The neural body of the cervical VI is complete; however, it contains an axial crack in the central part. The right postzygapophysis is partially preserved, whereas the left one is missing.

The cervical VIII (MMG CST 621; Fig. 16E) is almost complete as well as the largest vertebra of the vertebral series, in which both pre- and postzygapophyses are preserved. It is subsquare and has a relatively small anterior condyle. The posterior half of the vertebra is more than double in size compared to the anterior half one. A small concavity is preserved on the posterior edge of the left postzygapophysis.

**Pectoral girdle (syntypes)**. A complete pectoral girdle (MMUL 631/G 12912; Figs. 17A–17B) and a poorly preserved coracoid (MMUL 1461/G 12937; Fig. 17C) are preserved. The coracoid is the longest of the three pectoral processes that form the pectoral girdle: it is flat and wide, and its posterior margin is markedly curved. The angle between the coracoid and the scapular process is approximately 90°. Two elongated processes make up the scapula: the ventromedial process (acromion process) and the dorsal process (scapular process). The acromion process is shorter and is flat at its distal end. The scapular process is longer and wide, circular in cross section, and its distal end displays an articular surface. The glenoid cavity is heavily eroded and covered by matrix.

**Humerus**. An almost complete right humerus RMT PA 1684 (Figs. 17D–17E) is preserved, being only visible in ventral view. The shaft is slightly sinuous (S-shaped) and quite robust, as in all trionychids (Meylan, 1987). The proximal part of this bone, formed by the spherical humeral head and the major and minor trochanters, is wide. The major trochanter is well developed as it extends superiorly beyond the humeral head, whereas the minor trochanter is not preserved. The distal part of the humerus is also wide and asymmetric, as the ulnar condyle is larger than the radial.

**Pelvic girdle**. The left pubis and ischium RMT PA 1686 (Figs. 18A–18B) are preserved. The pubis is the most anterior element of the pelvic girdle; it is flat, smooth and sutured to the ischium forming the acetabulum. The lateral pubis process is missing and, thus, the anterior expansion of this process cannot be evaluated (Figs. 18A–18B). The anteromedial process is large, almost straight anteroposteriorly, and only displays a small notch in its posterior part. The posteromedial part of the pubis together with the ischium forms the thyroid fenestra, which is rounded and not divided by a pubioischiatic bridge. The acetabulum is almost hidden and provides no information about the glenoid cavity. The ischium forms the anterolateral edge of the thyroid fenestra and contributes significantly to the acetabulum (Figs. 18A–18B). The metischial process is present and developed in posterolateral direction. The anterior edge of the ischium is slightly convex (not straight) and the thelial process is absent.

**Femur**. Three femora are preserved. All of them resemble those of other trionychids appearance by having a longer than wide, sinuous shaft, and the absence of the open entepicondylar foramen in the distal epiphysis (Meylan, 1987). The proximal part of the femur is wide, with an elliptical femoral head oriented perpendicular to the shaft with a short neck (RMT PA 1683; Figs. 18C–18D). The major and minor trochanters are offset from the shaft; the former is more developed than the later and extends to about the same level as the femoral head (Figs. 18F–18G). All femoral shafts appear artificially slender as they are partially covered by matrix (Figs. 18C–18G). RMT PA 1308/1 (Figs. 18F–18G) shows that the tibial condyle is more developed than the fibular, and that the entepicondylar foramen is absent.

## Discussion

### Alpha-taxonomy

#### Nomenclatural and taxonomic remarks on Trionyx pontanus

The first mention of fossil trionychids from Czechia was made by Štúr (1874) from the Josef-Oswald mine in Tušimice (Most Basin), not far from Klášterec nad Ohří (see also Luján et al., 2019). Later, Laube (1895) defined the species *Trionyx pontanus* based on two individuals from Lom u Mostu. However, his work lacked detailed anatomical descriptions and figures. Soon thereafter, Laube (1896) provided a description based on previously mentioned two carapaces and their imprints (Fig. 2), highlighting the shape of the costals and last neurals, as well as the carapace sculpturing pattern. Bergounioux (1935) reported the presence of *Trionyx pontanus* from the island of Sardinia, but this was recently rejected by Georgalis & Joyce (2017). Comaschi Caria (1959) proposed a new combination of characters for Sardinian *T. pontanus* and he transferred it into the genus *Amyda* (see Georgalis et al., 2017). Karl (1998) considered all fossil *Trionyx* nominal species from Europe to be junior subjective synonyms of the extant African softshell turtle (*Trionyx triunguis*). Later, Chkhikvadze (1999) concluded that *T. pontanus* had been erected first, resurrecting it as the senior synonym of all species named from the Most Basin (*i.e., T*. *aspidiformis*, *T*. *bohemicus*, *T*. *elongatus*, and *T*. *preschenensis*) and further referring it to the genus *Rafetus* for the first time (*i*.*e*., *Rafetus pontanus*). Georgalis & Joyce (2017), in contrast, concluded that *T. pontanus* is a *nomen dubium* (a nomenclaturally available name of doubtful taxonomic application) as both syntypes are based exclusively on carapacial material, such that synonymy with *R*. *bohemicus* cannot be conclusively ascertained. It is noteworthy that our inspection of the syntype material of *T*. *pontanus* highlights several carapacial features diagnostic of the genus *Rafetus* (*i.e.,* the presence of seven neurals of which neural V is subsquare, a highly reduced costal VIII, and the absence of preneural plates). However, given that the plastral elements are not preserved for *T. pontanus* (*i.e*., the hyo- and hypoplastral callosities and the count of medial hyo- and hypoplastral processes cannot be evaluated), we concur with Georgalis & Joyce (2017) that *T. pontanus* is best considered a *nomen dubium*. In this regard, it is noteworthy that the type material of *T. pontanus* comes from different localities: one of the syntypes (RMGM G/pa77a and RMGM G/pa77b) comes from the Evžen (Eugen) Mine, Lom u Mostu (Bruch bei Brüx, in old literature), instead of Most (Brüx, in old literature) as erroneously reported by Georgalis & Joyce (2017). The other syntype (NMP Pv 12301) comes from the Pavel I Mine (Paulschacht), Louka u Litvínova (Wiese bei Leutensdorf, in old literature). In either case, both syntypes come from the Libkovice Member, Most Formation (Burdigalian, Early Miocene) and are slightly younger than the type material of *R. bohemicus* from Břešt’any (Novotný et al., 2021; Ekrt, Novotný & Přikryl, 2022).

#### Nomenclatural and taxonomic remarks on Trionyx preschenensis

Laube (1898) first used the name *T. preschnensis* for the Early Miocene (MN 3) material from Břeš’tany (Most Basin). However, the publication lacks formal criteria for its use as a scientific name nor the figures and proper description, *i.e*., it does not satisfy the requirements of the code (ICZN: International Commission on Zoological Nomenclature, 1999) to be nomenclaturally available for names published before 1931, and must be considered a *nomen nudum*. Shortly thereafter, Laube (1900) described the material in detail and figured the syntypes (an almost complete carapace and a right hypoplastron with its imprint), thus formally making the name available. Also, Laube (1900) defined the name as *Trionyx preschenensis*, instead of *Trionyx preschnensis*. Portis (1901) established *Procyclanorbis sardus* from the Miocene of Sardinia, and he considered *T*. *preschnensis* from Břešt’any to pertain also to the same genus, *Procyclanorbis* (see more in Georgalis et al., 2017). Karl (1998), in his revision of all fossil trionychids from Europe, considered *T. preschenensis* as a junior subjective synonym of *T*. *triunguis*. Chkhikvadze (1999) transferred *T*. *preschenensis* to the genus *Rafetus* and suggested that it was a junior synonym of *T*. *pontanus*. As recently noted by Georgalis & Joyce (2017), the syntypes of *T. preschenensis* (CUP CHMHZ–GL–0001 and NMP 20205/Pb 72) was initially housed at the Geological Institute of the German University in Prague, but later transferred to the NMP. However, only the imprint (NMP 20205/Pb 72; Figs. 4C–4D) was transferred to the National Museum in Prague, and the original was missing for several years (Figs. 4A–4B). Fortunately, one of the co-authors of this work (M.M.) found the original in the collections at Charles University in Prague (CUP). *T. preschenensis* was considered as *nomen dubium* by Georgalis & Joyce (2017), as it is based on a juvenile individual. Although our inspection of the hypoplastron (Figs. 4A–4B) allowed us to recognize some diagnostic features of the genus *Rafetus* (*e.g*., a single medial hypoplastral process and hypoplastron callosities restricted along the hyo-hypoplastral suture), we agree that, given the young ontogenetic stage and the fragmentary preservation of the holotype (most of the plastral elements are missing), it is not possible to provide an adequate diagnosis at the species level. Therefore, *Trionyx preschenensis* (Laube, 1900) is also considered a *nomen dubium*.

#### Nomenclatural and taxonomic remarks on Trionyx aspidiformis

Laube (1900) further named *Trionyx aspidiformis* based on an additional specimen (a partial carapace) from Břešt’any (Figs. 5A–5B, 6A–6D). The taxonomic validity of this nominal species was mostly ignored over the 19th century until Karl (1998) proposed that all Miocene European trionychids should be synonymized with the extant species *T. triunguis*. This proposal was not followed by Chkhikvadze (1999), as he argued that *T. aspidiformis* belongs to the genus *Rafetus* and is a junior synonym of *R. pontanus.* Recently, Georgalis & Joyce (2017) considered this taxon as a *nomen dubium* because it is based on a juvenile specimen. We here agree that both syntypes (NMP 36675/Pb 73, Figs. 5A–5B and RMT PA 1308/2, Figs. 6C–6D) of *T. aspidiformis* belong to a juvenile ontogenetic stage, as both individuals are small, and the costal plates are poorly developed medially. Based on our inspection of the type material, we conclude that *Trionyx aspidiformis* (Laube, 1900) is best considered a *nomen dubium*. In fact, the preserved morphology of both carapaces only allows a referral to the family rank, *i.e*., Trionychidae, in having a sculpting pattern that covers the carapace, and the absence of peripherals, pygals, suprapygals, and shell scutes.

#### Nomenclatural and taxonomic remarks on Trionyx elongatus

Liebus (1930) erected the third taxon from Břešt’any, *i.e., Trionyx elongatus*, from a small and elongated carapace and hypoplastron of a juvenile individual (NMP 1488/Pb 4; Figs. 7A–7B), and an isolated nuchal (MMUL 129/G 12911; Figs. 7C–7D). Liebus (1930) remarked the reduced number of neurals preserved in NMP 1488/Pb 4. Even though our inspection of the carapace did not allow to count all neural plates, as some sutures are obliterated, it is now known that the number of neurals is quite variable in both extant and extinct trionychids (*e.g.*, Meylan, 1987; Gardner & Russel, 1994). Karl (1998) synonymized *T. elongatus* with the extant *T*. *triunguis,* whereas Chkhikvadze (1999) considered it as a junior synonym of *R*. *pontanus*. Given that, *T. elongatus* was mainly based on a non-diagnostic juvenile individual (NMP 1488/Pb 4; Figs. 7A–7B), Georgalis & Joyce (2017) considered it to be a *nomen dubium*. We here agree with the taxonomical opinion of the previous authors*,* as the carapace length of NMP 1488/Pb 4 is rather small (∼11 cm) and the presence of laterally extended ribs also supports a juvenile stage. As for the isolated nuchal plate of the second individual (MMUL 129/G 12911; Figs. 7C–7D), the preserved morphology is also too limited to provide an adequate specific diagnosis and confirms that this nominal taxon should be considered a *nomen dubium*.

#### The genus assignment on Trionyx bohemicus

Liebus (1930) erected *Trionyx bohemicus* on the basis of material from the Early Miocene of Břešt’any (Most Basin, Czechia). Over the 20th century, this taxon was never revisited and most of the authors pay no attention, until Karl (1998) synonymized *T*. *bohemicus* with the extant *T*. *triunguis*. Chkhikvadze (1999) referred *T*. *bohemicus* to the genus *Rafetus* (*i.e., Rafetus pontanus*) based on a highly reduced plastron that lacks xiphiplastral callosities. This author considered the name *T. pontanus* valid as it had nomenclatural priority over *T. bohemicus*: therefore, *T. bohemicus* became a junior synonym of *T. pontanus*. More recently, Georgalis & Joyce (2017) concluded with Chkhikvadze (1999) that *T*. *bohemicus* is referable to the *Rafetus* lineage and considered it as the sole valid taxon among Czech trionychids. Due to the missing diagnostic plastral material of *R. pontanus*, *R. bohemicus* is the only taxonomically valid taxon from the Most Basin.

In accordance with Georgalis & Joyce (2017), our study reveals that *R*. *bohemicus* can be referred to the family Trionychidae based on the following features: a quadratojugal that is not in contact with the postorbital or maxilla; exclusion of the premaxillae from the external naris; presence of ornamentation that covers all metaplastic portions of the shell bones (*i.e.,* carapace and plastron plates); the absence of peripherals, pygals and suprapygals; boomerang-shaped entoplastron; and the absence of central articulation between the eighth cervical and the first thoracic vertebrae (Georgalis & Joyce, 2017).

Our inspection of available shell material of *Trionyx bohemicus* from the Most Basin allowed us to refer the material to the genus *Rafetus*, based on the following characters: absence of preneural plate, seven neurals of which neural V is rectangular (longer than wide), highly reduced costals plates VIII, a reduced number of medial hyo- and hypoplastral processes, and poorly developed callosities restricted to the hyo-hypoplastron (Georgalis & Joyce, 2017). As for the skulls, we can confidently refer them to the genus *Rafetus* based on the following features: a shorter and broader snout (instead of long and narrow as in *Trionyx vindobonensis*), a non-concave medial edge of the maxilla in palatal view (instead of straight), a short intermaxillary suture developed between the intermaxillary foramen and internal naris (instead of long), and a large internal naris (instead of medium or small-sized).

Two different extant species of the genus *Rafetus* are currently considered taxonomically valid: *Rafetus euphraticus* (Daudin, 1801), which is the type species of the genus, found throughout much of the Euphrates-Tigris River in Iran, Irak, Syria and Turkey; and *Rafetus swinhoei* (Gray, 1873), currently distributed in China and Vietnam (TTWG: Turtle Taxonomy Working Group et al., 2021). According to Georgalis & Joyce (2017), *R. bohemicus* differs from *R. euphraticus* in having a more pronounced mediolateral constriction of the xiphiplastral elements, and from *R. swinhoei* in having a smaller shell. As for the skull, Meylan (1987) noted that the difference between the two extant species was the contact between basisphenoid and palatines, being reduced or absent in *R. euphraticus*, and broadly developed in *R. swinhoei*. However, Taşkavak (1999) showed that this contact is quite variable among *Rafetus* species so that, for instance, in *R. euphraticus* all three states can be displayed, *i.e*., absent, reduced, or broadly developed.

**Figure 19:**
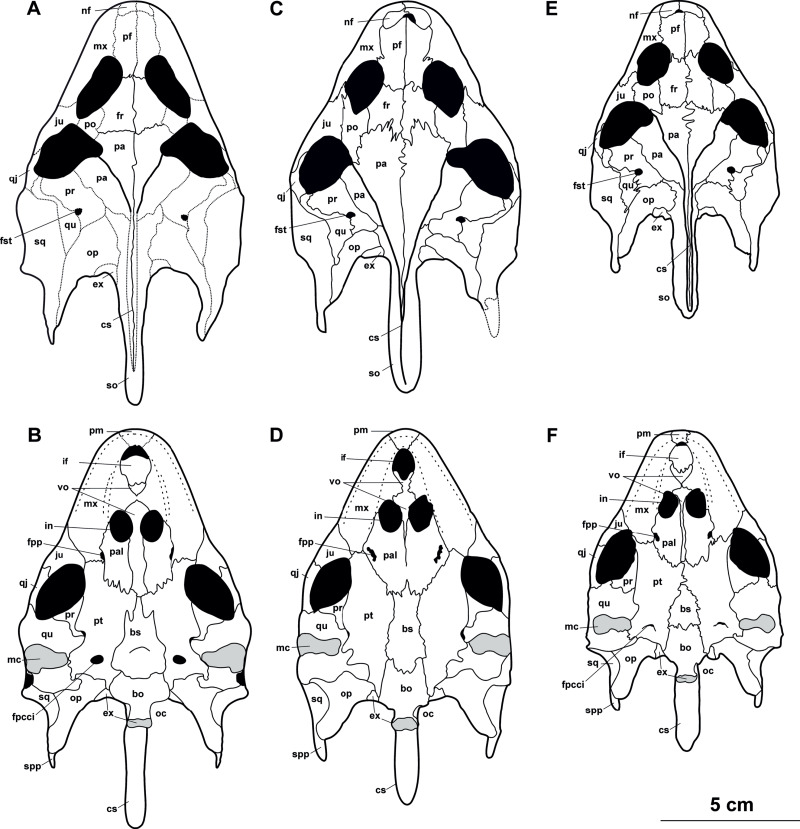
The reconstruction of skulls of *Rafetus* species. *Rafetus bohemicus* based on the available cranial bones in dorsal (A) and visceral (B) views. *Rafetus swinhoei* IEBRH NQT85 in dorsal (C) and NHMUK 1947.3.6.13 visceral (D) views. *Rafetus euphraticus* NHMW 130 in dorsal (E) and visceral (F) views. Abbreviations: bo, basioccipital; bs, basisphenoid; cs, crista supraoccipitalis; exo, exoccipital; fpcci, foramen posterius canalis carotici interni; fpp, foramen palatinum posterius; fr, frontal; fst, fst, foramen stapedio-temporale; if, intermaxillary foramen; in, internal naris; ju, jugal; mc, mandibular condyle; mx, maxilla; nf, nasal fossa; oc, occipital condyle; op, opisthotic; pa, parietal; pal, palatine; pf, prefrontal; pm, premaxilla; po, postorbital; pr, prootic; pt, pterygoid; pr, prootic; qj, quadratojugal; qu, quadrate; spp, squamosal posterior processes; sq, squamosal; vo, vomer. The discontinuous lines denote the hypothetical sutures.

Our virtual 3D models of the two selected skulls derived from CT scans (Figs. 8B–8C, 9A–9B, 12B) allow us to ascertain additional morphological details of *R. bohemicus*. The premaxillae can be clearly observed in both NMP Pv 11668 (Figs. 9A–9B) and MMUL 1037/G 12917 (Fig. 12B), being very narrow, but seemingly participating in the intermaxillary foramen. Among *Rafetus* species, this condition is displayed by the two extant species, but in *R. bohemicus* it cannot be confidently ascertained (the dotted line in Fig. 19A). One of the described specimens from Břešt’any (NMP Pv 11668, Figs. 9A–9B) preserves most of the vomer, a long element that forms the posterior edge of the intermaxillary foramen and can be divided medially by the maxillae, just between the internal nares and the intermaxillary foramen. The basisphenoid can only be evaluated in MMUL 1048/G 10194 (Figs. 12D–12E), being subtriangular and contacting with the vomer anteromedially, but not the palatines. Direct inspection of both extant *Rafetus* species revealed that this condition is present in *R. euphraticus* (NHMW 130, Fig. 19C), although the contact is quite variable, so that the vomer can also separate the maxillae (*e.g*., Farkas & Fritz, 1998; NHMW 131, Fig. 2C; Taşkavak, 1999; ZDEU-67/990-4, Fig. 6B). The foramen palatinum posterius, only visible in NMP Pv 11668 (Figs. 9B–9C), is located on the posterior portion of the maxilla and palatinum, *i.e.,* the pterygoid is excluded. Although, slightly more located on the palatine, *R. euphraticus* displays a similar condition to *R. bohemicus* (Fig. 19C), whereas *in R. swinhoei* the foramen palatinum posterius is completely located within the pterygoid (Fig. 19B).

As for postcranial elements, a very short dorsal process of the pectoral girdle (acromion process), with a round distal end, is found in *R. bohemicus* (MMUL 631/G 12912, Figs. 17A–17B). Thus, the material from Břešt’any differs from *R. euphraticus* and *R. swinhoei*, in which the acromion process is long, and the distal end is subrectangular (Ahranjani et al., 2016). Lastly, regarding the plastral elements, the solely available hyoplastron (MMUL I/G 1486/G 12939, Figs. 15A–15B) shows single (undivided) anterolateral hyoplastral process. According to published data (*e.g.*, Siebenrock, 1913; Bettelheim, 2012; Berthon et al., 2016), in the two extant species of *Rafetus* the anterolateral hyoplastral process shows fingering, *i.e.,* its distal end is always divided into two processes. In summary, the revision of all specimens from Břešt’any (MN 3), which include skull, plastral, and postcranial elements, allows us confidently to differentiate *Rafetus bohemicus* from the rest of extant *Rafetus* species.

### Evolutionary and paleobiogeographic implications

Phylogenetic analyses performed by Le et al. (2014, Fig. 5) dated the divergence between extant *Rafetus* species (*R. swinhoei* and *R. euphraticus*) to the Early Miocene (∼20 Ma). *Rafetus bohemicus* from Břešt’any is just slightly younger (∼17.5 Ma) than this divergence date and represents the first appearance of this genus in the world and, surprisingly, Europe, coinciding with the onset of the global warming period known as the Mid-Miocene Climatic Optimum (Zachos et al., 2001). The Mid-Miocene Climatic Optimum is well documented in the Most Basin, starting during the transition between of the Holešice and Libkovice formations (Teodoridis & Kvaček, 2006; Matys Grygar et al., 2014; Matys Grygar et al., 2017a). Despite this, more fossil data from Europe and Asia would be required to unravel the paleobiogeographical history of *Rafetus* species. Apparently, *R*. *bohemicus* inhabited Central and Eastern Europe and its geographic distribution being restricted by the Central Paratethys Sea and by the Carpathian Mountain Range to the south, whereas *Trionyx vindobonensis* occupied Western, Southern, and partly Central Europe (Georgalis & Joyce, 2017).

## Conclusions

We provide a revision and emended diagnosis of the extinct trionychid species *Rafetus bohemicus* based on the currently available material (cranial, shell, and postcranial remains) from the Most Basin, Czechia (MN 3, Burdigalian, Early Miocene). The large sample studied in this work, including material mainly from Břešt’any, confirms that the nominal taxa *Trionyx pontanus*, *T*. *preschenensis*, *T*. *aspidiformis*, and *T*. *elongatus* are nomina dubia. Our descriptions highlight new diagnostic cranial characters that allowed us to emend the diagnosis of *Rafetus bohemicus* and which enable a better differentiation of trionychid taxa from the Early Miocene of Europe.

##  Supplemental Information

10.7717/peerj.15658/supp-1 Supplemental Information 1 Skull of *Rafetus bohemicus* The 3D visualization of the skull of *Rafetus bohemicus* (NMP Pv 11668). 3D model credit: Michal Vopálenský.

## Abbreviations

CUP: Chlupáč’s Museum of Earth History at Charles University in Prague, Czechia
IEBRH: Institute of Ecology and Biological Resources in Hanoi, Vietnam
IPUW: Institute of Palaeontology at University of Vienna, Austria
MMG: Museum of Mineralogy and Geology in Dresden, Germany
MMUL: Municipal Museum of Ústí nad Labem, Czechia
NHMUK: Natural History Museum in London, UK
NHMW: Natural History Museum in Vienna, Austria
NMP: National Museum in Prague, Czechia
RMGM: Regional Museum and Gallery of Most, Czechia
RMT: Regional Museum of Teplice, Czechia

## Additional Information and Declarations

### Field Study Permissions

The following information was supplied relating to field study approvals (i.e., approving body and any reference numbers): CUP, Chlupáč’s Museum of Earth History at Charles University in Prague, Czechia; IEBRH, Institute of Ecology and Biological Resources in Hanoi, Vietnam; IPUW, Institute of Palaeontology at University of Vienna, Austria; MMG, Museum of Mineralogy and Geology in Dresden, Germany; MMUL, Municipal Museum of Ústí nad Labem, Czechia; NHMUK, Natural History Museum in London, UK; NHMW, Natural History Museum in Vienna, Austria; NMP, National Museum in Prague, Czechia; RMGM, Regional Museum and Gallery of Most, Czechia; RMT, Regional Museum of Teplice, Czechia.

### Data Availability

The following information was supplied regarding data availability: The NMP:PV:11668 Rafetus bohemicus is available at MorphoSource: - https://doi.org/10.17602/M2/M469442 (CT Image Series) - https://doi.org/10.17602/M2/M474314 (mesh file derivative of CT Image Series 000469442) - https://doi.org/10.17602/M2/M459259 (video animation file derivative of CT Image Series 000469442).

## References

[ref-1] Aguilar J-P, Legendre S, Michaux J (1997). Actes du Congrès Biochrom ’97, Montpellier, 14–17 avril: biochronologie mammalienne du cénozoïque en Europe et domaines reliés [Proceedings of the Biochrom ’97 Congress, Montpellier, April 14–17: Cenozoic mammalian biochronology in Europe and related fields.].

[ref-2] Ahranjani BA, Shojaei B, Tootian Z, Masoudifard M, Rostami A (2016). Anatomical, radiographical and computed tomographic study of the limbs skeleton of the Euphrates soft shell turtle (*Rafetus euphraticus*). Veterinary Research Forum.

[ref-3] Batsch AJGC (1788). Versuch einer Anleitung zur Kenntniss und Geschichte der Thiere und Mineralien. Erster Theil. Allgemeine Geschichte der Natur; besondre der Säugthiere, Vögel, Amphibien und Fische [Attempt at a guide to the knowledge and history of animals and minerals. First part. general history of nature; especially the mammals, birds, amphibians and fish.].

[ref-4] Bell T (1828). Characters of the order, and families, and genera of the Testudinata. Zoological Journal.

[ref-5] Bergounioux FM (1935). Contribution à l’étude paléontologique des chéloniens: chéloniens fossils du basin d’Aquitaine [Contribution to the paleontological study of chelonians: fossil chelonians from the Aquitaine basin]. Mémoires de la Société Géologique De France.

[ref-6] Berthon R, Erdal YS, Mashkour M, Kozbe G (2016). Buried with turtles: the symbolic role of the Euphrates soft-shelled turtle (*Rafetus euphraticus*) in Mesopotamia. Antiquity.

[ref-7] Bettelheim MP (2012). Swinhoe’s softshell turtle (*Rafetus swinhoei*): the legendary sword lake turtle of Hoan Kiem Lake. Bibliotheca Herpetologica.

[ref-8] Cadena ER, Bourgue JR, Rincon AF, Bloch JI, Jaramillo CA, MacFadden BJ (2012). New turtles (Chelonia) from the late Eocene through late Miocene of the Panama Canal Basin. Journal of Paleontology.

[ref-9] Chkhikvadze VM (1999). Some fossil soft-shelled turtles of Asia (Rafetini trib, nov.). Trudy Tbilisskogo Gosudarstvennogo Pedagogicheskogo Universiteta.

[ref-10] Comaschi Caria J (1959). Nuovi resti di cheloni nel Miocene della Sardegna. Bollettino Della Società Geologica Italiana.

[ref-11] Cope ED (1868). On the Origin of Genera. Proceedings of the Academy of Natural Sciences of Philadelphia.

[ref-12] Daudin FM (1801). Histoire naturelle, générale et particulière, des reptiles: ouvrage faisant suite à l’Histoire naturelle générale et particulière, composée par Leclerc De Buffon, et rédigée par C.S. Sonnini [Natural history, general and particular, of the reptiles: work making following the General and Particular Natural History, composed by Leclerc De Buffon, and written by C.S. Sonnini.].

[ref-13] Delfino M, Scheyer TM, Fritz U, Sánchez-Villagra MR (2010). An integrative approach to examining a homology question: shell structures in soft-shell turtles. Biological Journal of the Linnean Society.

[ref-14] Dvořák Z, Mach K, Prokop J, Knor S (2010). Třetihorní fauna severočeské hnědouhelné pánve.

[ref-15] Ekrt B, Novotný T, Přikryl T (2022). New ichthyofauna from the Holešice and Libkovice members in the western part of Most Basin (Early Miocene), the Czech Republic. Fossil Imprint.

[ref-16] Engstrom TN, Shaffer HB, McCord WP (2004). Multiple data sets, high homoplasy, and the phylogeny of softshell turtles (Testudines: Trionychidae). Systematic Biology.

[ref-17] Farkas B, Fritz U (1998). On the identity of *Rafetus swinhoei* (Gray, 1873) and *Pelochelys maculatus* (Heude, 1880) (Reptilia: Testudines: Trionychidae). Zoologische Abhandlungen Staatliches Museum für Tierkunde Dresden.

[ref-18] Fejfar O, Lindsay EH, Fahlbusch V, Mein P (1989). The neogene VP sites of Czechoslovakia: a contribution to the neogene terrestric biostratigraphy of europe based on rodents. European neogene mammal chronology.

[ref-19] Fejfar O, Kvaček Z (1993). Excursion (3) Tertiary basins in Northwest Bohemia.

[ref-20] Fejfar O, Dvořák Z, Kadlecová E, Reumer JWF, Wessels W (2003). New record of Early Miocene (MN3a) mammals in the open brown coal pit Merkur, North Bohemia, Czech Republic. Distribution and migration of Tertiary mammals in Eurasia. A volume in honour of Hans De Bruijn.

[ref-21] Fejfar O, Sabol M, Van den Hoek Ostende LW, Doukas CS, Reumer JWF (2005). Czech Republic and Slovak Republic. The fossil record of the Eurasian neogene insectivores Erinaceomorpha, Soricomorpha, Mammalia, Part I.

[ref-22] Forskål P (1775). Descriptiones animalium, and avium, and amphibiorum, and piscium, and insectorum, vermium quae in itinere orientali observavit.

[ref-23] Fucini A (1912). *Trionyx pliocenicus* Law. Paleontographia Italica.

[ref-24] Gaffney ES (1972). An illustrated glossary of turtle skull nomenclature. American Museum Novitates.

[ref-25] Gaffney ES (1979). Comparative cranial morphology of recent and fossil turtles. Bulletin of the American Museum of Natural History.

[ref-26] Gardner JD, Russel AP (1994). Carapacial variation among soft-shelled turtles (Testudines: Trionychidae), and its relevance to taxonomic and systematic studies of fossil taxa. Neues Jahrbuch für Geologie und Paläontologie—Abhandlungen.

[ref-27] Geoffroy Saint-Hilaire EF (1809). Mémoire sur les tortues molles, nouveau genre sous le nom de *Trionyx*, et sur la formation des carapaces. Annales du Muséum d’Histoire Naturelle.

[ref-28] Georgalis GL (2021). First pan-trionychid turtle (Testudines, Pan-Trionychidae) from the Palaeogene of Africa. Papers in Palaeontology.

[ref-29] Georgalis GL, Joyce WG (2017). A review of the fossil record of Old World turtles of the clade *Pan-Trionychidae*. Bulletin of the Peabody Museum of Natural History.

[ref-30] Georgalis GL, Zoboli D, Pillola GL, Delfino M (2017). A revision of the trionychid turtle *Procyclanorbis sardus* Portis, 1901 from the late Miocene of Sardinia (Italy). Annales de Paléontologie.

[ref-31] Georgalis GL, Insacco G, Rook L, Spadola F, Delfino M (2020). Turtle remains from the late Miocene of the Cessaniti area, southern Italy—insights for a probable Tortonian chelonian dispersal from Europe to Africa. Swiss Journal of Palaeontology.

[ref-32] Gray JE (1864). Revision of the species of Trionychidae found in Asia and Africa, with the description of some new species. Proceedings of the Zoological Society of London.

[ref-33] Gray JE (1873). Notes on Chinese mud-tortoises (Trionychidae), with the description of a new species sent to the British Museum by Mr. Swinhoe, and observations on the male organ of this family. The Annals and Magazine of Natural History; Zoology, Botany, and Geology.

[ref-34] Head JJ, Aguilera OA, Sánchez-Villagra MR (2006). Past colonization of South America by trionychid turtles: fossil evidence from the Neogene of Margarita Island, Venezuela. Journal of Herpetology.

[ref-35] Hirayama R, Isaji S, Hibino T, Brinkman D, Holroyd P, Gardner J (2013). *Kappachelys okurai* gen. et sp. nov. a New Stem Soft-Shelled Turtle from the Early Cretaceous of Japan. Morphology and Evolution of Turtles.

[ref-36] ICZN: International Commission on Zoological Nomenclature (1999). International code of zoological nomenclature.

[ref-37] Joyce WG, Anquetin J, Cadena EA, Claude J, Danilov IG, Evers SW, Ferreira GS, Gentry AD, Georgalis GL, Lyson TR, Pérez-García A, Rabi M, Sterli J, Vitek NS, Parham JF (2021). A nomenclature for fossil and living turtles using phylogenetically defined clade names. Swiss Journal of Palaeontology.

[ref-38] Karl H-V (1998). Zur Taxonomie der känozoischen Weichschildkröten Österreichs und Deutschlands (Trionychidae: Trionychinae) [On the taxonomy of the Austrian Cenozoic softshell turtles and Germany (Trionychidae: Trionychinae).]. Mitteilungen Zur Geologie und Paläontologie, Land Esmuseum Joanneum.

[ref-39] Kvaček Z, Böhme M, Dvořák Z, Konzalová M, Mach K, Prokop J, Rajchl M (2004). Early Miocene freshwater and swamp ecosystems of the Most Basin (northern Bohemia) with particular reference to the Bílina Mine section. Journal of the Czech Geological Society.

[ref-40] Laube CG (1895). Vorläufiger Bericht über Schildkrötenreste aus der böhmischen Braunkohlenformation [Preliminary report on tortoise remains from the Bohemian lignite formation]. Verhandlungen der Geologischen Bundesanstalt.

[ref-41] Laube CG (1896). Schildkrötenreste aus der böhmischen Braunkohlenformation [Turtle remains from the Bohemian lignite formation]. Abhandlungen des Deutschen Naturwissenschaftlich-Medizinischen Vereins für Böhmen “Lotos”.

[ref-42] Laube CG (1898). Bericht über einen neuen *Trionyx* aus den aquitanischen (plastischen) Thonen von Preschen bei Bilin in Böhmen [Report of a New Trionyx from the Aquitanian (Plastic) Thonen from Preschen near Bilin in Bohemia]. Verhandlungen der Geologischen Bundesanstalt.

[ref-43] Laube CG (1900). Neue Schildkröten und Fische aus der böhmischen Braunkohlenformation [New turtles and fish from the Bohemian lignite formation.]. Abhandlungen des Deutschen Naturwissenschaftlich-Medizinischen Vereins für Böhmen “Lotos”.

[ref-44] Laube CG (1901). Synopsis der Wirbeltierfauna der böhmischen Braunkohlenformation und Beschreibung neuer, oder bisher unvollständig bekannter Arten [Synopsis of the vertebrate fauna of the Bohemian lignite formation and description of new or hitherto incompletely known species]. Lotos.

[ref-45] Le M, Duong HT, Dinh LD, Nguyen TQ, Pritchard PCH, McCormack T (2014). A phylogeny of softshell turtles (Testudines: Trionychidae) with reference to the taxonomic status of the critically endangered, giant softshell turtle, *Rafetus swinhoei*. Organisms Diversity and Evolution.

[ref-46] Li H, Liu J, Xiong L, Zhang H, Zhou H, Yin H, Jing W, Li J, Shi Q, Wang Y, Liu J, Nie L (2017). Phylogenetic relationships and divergence dates of softshell turtles (Testudines: Trionychidae) inferred from complete mitochondrial genomes. Journal of Evolutionary Biology.

[ref-47] Li L, Joyce WG, Liu J (2015). The first soft-shelled turtle from the Jehol Biota of China. Journal of Vertebrate Paleontology.

[ref-48] Li L, Tong H, Gu W, Liu J (2015). A new trionychid turtle from the Early Cretaceous of Heilongjiang Province, Northeastern China. Cretaceous Research.

[ref-49] Liebus A (1930). Neue Schildkröten aus den Tertiären Süsswassertonen von Preschen bei Bilin in Böhmen [New turtles from the Tertiary freshwater clays of Preschen Bilin in Bohemia]. Rozpravy Státního geologického ústavu Československé Republiky.

[ref-50] Luján ÀH, Chroust M, Čerňanský A, Fortuny J, Mazuch M, Ivanov M (2019). First record of *Diplocynodon ratelii* Pomel, 1847 from the early Miocene site of Tušimice (Most Basin, Northwest Bohemia, Czech Republic). Comptes Rendus Palevol.

[ref-51] Lydekker R (1889). Catalogue of the fossil Reptilia and Amphibia in the British Museum. Part III. Chelonia.

[ref-52] Mach K, Teodoridis V, Grygar TM, Kvaček Z, Suhr P, Standke G (2014). An evaluation of palaeogeography and palaeoecology in the Most Basin (Czech Republic) and Saxony (Germany) from the late Oligocene to the early Miocene. Neues Jahrbuch für Geologie und Paläontologie—Abhandlungen.

[ref-53] Mach K, Žák K, Teodoridis V, Kvaček Z (2017). Consequences of Lower Miocene CO_2_ degassing on geological and paleoenvironmental settings of the Ahníkov/Merkur Mine paleontological locality (Most Basin, Czech Republic). Neues Jahrbuch für Geologie und Paläontologie—Abhandlungen.

[ref-54] Matys Grygar T, Mach K (2013). Regional chemostratigraphic key horizons in the macrofossil-barren siliciclastic lower Miocene lacustrine sediments (Most Basin, Eger Graben, Czech Republic). Bulletin of Geosciences.

[ref-55] Matys Grygar T, Mach K, Schnabl P, Pruner P, Laurin J, Martinez M (2014). A lacustrine record of the early stage of the Miocene Climatic Optimum in Central Europe from the Most Basin, Ohře (Eger) Graben, Czech Republic. Geological Magazine.

[ref-56] Matys Grygar T, Hošek M, Mach K, Schnabl P, Martinez M (2017a). Climatic instability before the Miocene Climatic Optimum reflected in a Central European lacustrine record from the Most Basin in the Czech Republic. Palaeogeography, Palaeoclimatology, Palaeoecology.

[ref-57] Matys Grygar T, Mach K, Hošek M, Schnabl P, Martinez M, Koubová M (2017b). Early stages of clastic deposition in the Most Basin (Ohře rift, Czech Republic, Early Miocene): timing and possible controls. Bulletin of Geosciences.

[ref-58] Meylan PA (1987). The phylogenetic relationships of soft-shelled turtles (family Trionychidae). Bulletin of the American Museum of Natural History.

[ref-59] Nessov L (1995). On some Mesozoic turtles of the Fergana depression (Kyrgyzstan) and Dzhungar Alatau Ridge (Kazakhstan). Russian Journal of Herpetology.

[ref-60] Novotný T, Teodoridis V, Kvaček Z, Váňa J, Mach K (2021). New floras of the Libkovice Member from the Libouš Mine (early Miocene, Most Basin). Geoscience Research Reports.

[ref-61] Peters KF (1855). Schildkrötenreste aus den österreichischen Tertiär-Ablagerungen [Turtle remains from the Austrian Tertiary deposits.]. Denkschriften der Kaiserlichen Akademie der Wissenschaften, Mathematisch-Naturwissenschaftliche Classe.

[ref-62] Portis A (1901). Il *Procyclanorbis sardus* Port. nuovo trionychide fossile della Sardegna [Procyclanorbis sardus Port. new fossil trionychid from Sardinia.]. Bollettino Della Società Geologica Italiana.

[ref-63] Rajchl M, Uličný D, Grygar R, Mach K (2009). Evolution of basin architecture in an incipient continental rift: the Cenozoic Most Basin, Eger Graben (Central Europe). Basin Research.

[ref-64] Sánchez-Villagra MR, Asher RJ, Rincón AD, Carlini AA, Meylan PA, Purdy RW, Sánchez-Villagra MR, Clack JA (2004). New faunal reports for the Cerro la Cruz locality (Lower Miocene), north-western Venezuela. Fossils of the Miocene Castillo Formation, Venezuela: Contributions on neotropical palaeontology.

[ref-65] Scheyer TM, Mörs T, Einarsson E (2012). First record of soft-shelled turtles (Cryptodira, Trionychidae) from the Late Cretaceous of Europe. Journal of Vertebrate Paleontology.

[ref-66] Selvatti AP, Moreira FRR, Carvalho DC, Prosdocimi F, De M Russo CA, Junqueira ACM (2023). Phylogenomics reconciles molecular data with the rich fossil record on the origin of living turtles. Molecular Phylogenetics and Evolution.

[ref-67] Siebenrock KF (1913). Schildkröten aus Syrien und Mesopotamien [Tortoises from Syria and Mesopotamia.]. Annalen des Naturhistorischen Museums in Wien.

[ref-68] Štúr D (1874). Trionyx und andere Petrefakten aus der Braunkohle von Klösterle [Trionyx and other petrefacts from the Klösterle lignite.]. Verhandlungen der Kaiserlich-Königlichen Geologischen Reichsanstalt.

[ref-69] Taşkavak E (1999). Cranial morphology of *Rafetus euphraticus* (Daudin, 1801) from Southeastern Anatolia. Amphibia-Reptilia.

[ref-70] Teodoridis V, Kvaček Z (2006). Palaeobotanical research of the Early Miocene deposits overlying the main coal seam (Libkovice and Lom Members) in the Most Basin (Czech Republic). Bulletin of Geosciences.

[ref-71] Rhodin AGJ, Iverson JB, Bour R, Fritz U, Georges A, Shaffer HB, Van Dijk PP, TTWG: Turtle Taxonomy Working Group (2021). Turtles of the world: annotated checklist and atlas of taxonomy, synonymy, distribution, and conservation status.

[ref-72] Van den Hoek Ostende LW, Fejfar O (2006). Erinaceidae and Talpidae (Erinaceomorpha, Soricomorpha, Mammalia) from the Lower Miocene of Merkur-Nord (Czech Republic, MN 3). Beiträge zur Paläontologie.

[ref-73] Van den Hoek Ostende LW, Fejfar O (2015). All time high: Dimylidae (Eulipotyphla, Mammalia) diversity in the early Miocene locality of Ahníkov 1 (Czech Republic, MN 3). Palaeobiodiversity and Palaeoenvironments.

[ref-74] Vitek NS, Danilov IG (2010). New material and a reassessment of soft-shelled turtles (Trionychidae) from the Late Cretaceous of Middle Asia and Kazakhstan. Journal of Vertebrate Paleontology.

[ref-75] Vitek NS, Joyce WG (2015). A review of the fossil record of new world turtles of the clade *Pan-Trionychidae*. Bulletin of the Peabody Museum of Natural History.

[ref-76] Zachos JC, Pagani M, Sloan L, Thomas E, Billups K (2001). Trends, rhythms, and aberrations in global climate 65 Ma to Present. Science.

[ref-77] Zangerl R, Gans G, Bellasirs AD (1969). The turtle shell. The biology of the Reptilia.

